# Sequence-based GWAS, network and pathway analyses reveal genes co-associated with milk cheese-making properties and milk composition in Montbéliarde cows

**DOI:** 10.1186/s12711-019-0473-7

**Published:** 2019-07-01

**Authors:** Marie-Pierre Sanchez, Yuliaxis Ramayo-Caldas, Valérie Wolf, Cécile Laithier, Mohammed El Jabri, Alexis Michenet, Mekki Boussaha, Sébastien Taussat, Sébastien Fritz, Agnès Delacroix-Buchet, Mickaël Brochard, Didier Boichard

**Affiliations:** 1 GABI, INRA, AgroParisTech, Université Paris Saclay, 78350 Jouy-en-Josas, France; 2 Conseil Elevage 25-90, 25640 Roulans, France; 3 Institut de l’Elevage, 75012 Paris, France; 4 Allice, 75012 Paris, France; 5 Umotest, 01250 Ceyzériat, France

## Abstract

**Background:**

Milk quality in dairy cattle is routinely assessed via analysis of mid-infrared (MIR) spectra; this approach can also be used to predict the milk’s cheese-making properties (CMP) and composition. When this method of high-throughput phenotyping is combined with efficient imputations of whole-genome sequence data from cows’ genotyping data, it provides a unique and powerful framework with which to carry out genomic analyses. The goal of this study was to use this approach to identify genes and gene networks associated with milk CMP and composition in the Montbéliarde breed.

**Results:**

Milk cheese yields, coagulation traits, milk pH and contents of proteins, fatty acids, minerals, citrate, and lactose were predicted from MIR spectra. Thirty-six phenotypes from primiparous Montbéliarde cows (1,442,371 test-day records from 189,817 cows) were adjusted for non-genetic effects and averaged per cow. 50 K genotypes, which were available for a subset of 19,586 cows, were imputed at the sequence level using Run6 of the 1000 Bull Genomes Project (comprising 2333 animals). The individual effects of 8.5 million variants were evaluated in a genome-wide association study (GWAS) which led to the detection of 59 QTL regions, most of which had highly significant effects on CMP and milk composition. The results of the GWAS were further subjected to an association weight matrix and the partial correlation and information theory approach and we identified a set of 736 co-associated genes. Among these, the well-known caseins, *PAEP* and *DGAT1*, together with dozens of other genes such as *SLC37A1*, *ALPL*, *MGST1*, *SEL1L3*, *GPT*, *BRI3BP*, *SCD*, *GPAT4*, *FASN*, and *ANKH*, explained from 12 to 30% of the phenotypic variance of CMP traits. We were further able to identify metabolic pathways (e.g., phosphate and phospholipid metabolism and inorganic anion transport) and key regulator genes, such as *PPARA*, *ASXL3,* and *bta*-*mir*-*200c* that are functionally linked to milk composition.

**Conclusions:**

By using an approach that integrated GWAS with network and pathway analyses at the whole-genome sequence level, we propose candidate variants that explain a substantial proportion of the phenotypic variance of CMP traits and could thus be included in genomic evaluation models to improve milk CMP in Montbéliarde cows.

**Electronic supplementary material:**

The online version of this article (10.1186/s12711-019-0473-7) contains supplementary material, which is available to authorized users.

## Background

About 40% of the bovine milk produced worldwide is processed into cheese; because of this, the cheese-making properties (CMP) of bovine milk are economically important for the dairy industry. Direct measurement of CMP is costly and time-consuming, and cannot be obtained on a very large scale. However, mid-infrared (MIR) spectrometry, which is already widely employed to predict milk composition, has been shown to provide indirect measures of CMP that are sufficiently reliable to be used in genetic analyses [1]. Indeed, because of their strong dependence on milk composition traits [2], milk CMP, especially cheese yields and coagulation properties, can be routinely assessed at low cost from MIR spectra [3]. The information obtained from high-throughput MIR spectra can then be combined with genotypic data from cows that are generated for the purpose of genomic selection to provide a unique resource for large-scale genomic analyses of CMP aimed at identifying the genes involved in the genetic determinism of these traits.

Genomic regions containing quantitative trait loci (QTL) that affect traits of interest, such as CMP, can be identified by genome-wide association studies (GWAS). By combining the results of genotyping for genomic selection with reference data from the 1000 Bull Genomes Project, it becomes possible to carry out GWAS on imputed whole-genome sequences (WGS) that should contain the causative mutations for traits of interest [4]. However, even if these analyses are carried out at the sequence level, GWAS alone is generally not sufficient to identify causative genes, let alone causative variants for complex and polygenic traits. Indeed, due to the long-range linkage disequilibrium (LD) in dairy cattle, many variants with almost identical P-values that are potentially located in more than one gene or in intergenic regions are generally found in a QTL region, which complicates identification of the causative mutations. Moreover, complex traits are typically influenced by many genomic regions, most of which explain only a small proportion of the phenotypic variance and are thus difficult to detect by GWAS. Finally, GWAS performed on a single trait and single marker cannot take either the pleiotropic effects of variants or the interactions between them into account. Thus, a GWAS-based approach is a good starting point for identifying QTL regions but needs to be supplemented by additional analyses to capture a larger proportion of the genetic variance and to understand in depth the genetic architecture of complex traits, such as CMP. In the last decade, methods have been developed that build on GWAS results by using gene network analysis to highlight co-associated genes for a set of correlated traits [5, 6]. Once the gene network is built, it is then possible to carry out in silico functional analyses, based on databases from bovine or other organisms’ genomes, to identify key regulators that modulate gene expression or to highlight the enrichment of gene-sets linked to certain metabolic pathways. Gene network approaches have been applied to milk CMP [7], fatty acid composition [8, 9], and protein composition [10, 11] but, to date, there has been no joint analysis of CMP and milk composition in spite of the close relationship between the two groups of traits. Moreover, all previous studies examined only a limited number of cows (164 to 1100 cows) and genomic variants (50 K or HD SNP chips).

The goal of the FROM’MIR project is to analyze CMP and milk composition traits predicted from MIR spectra in the Montbéliarde dairy breed from the Franche-Comté region, which boasts the highest production of protected designation of origin (PDO) cheeses in France. Nine CMP traits (three measures of cheese yield, five coagulation traits, and one acidification trait) and 27 milk composition traits (protein, fatty acid, mineral, citrate, and lactose contents) were predicted with a relatively high degree of accuracy from more than 6.6 million MIR spectra of milk samples collected from 410,622 cows. Of these cows, 19,586 were genotyped with a SNP chip. A prior study revealed medium-to-high heritabilities for CMP traits as well as high genetic correlations among CMP traits and between CMP and some milk composition traits [3].

The objectives of the current study were first, to fine-map QTL for CMP and milk composition traits via GWAS of imputed WGS from 19,586 cows, and second, to explore the GWAS results using association weight matrices (AWM) [5] and partial correlation and information theory (PCIT) [6] analyses, in order to identify gene networks and metabolic and regulatory pathways that are associated with milk cheese-making and composition traits.

## Methods

### Animals, MIR spectra, and 50 K genotypes

For this study, we did not perform any experiments on animals; thus, no ethical approval was required. Details of the animals, milk analyses, and prediction equations were described in a prior study by Sanchez et al. [3]. Briefly, prediction equations were developed for nine CMP traits from 416 milk samples for which both reference measurements for those CMP traits and MIR spectra were taken. The CMP traits, described in Table 1, included three laboratory cheese yields (CY_FRESH_, CY_DM_, and CY_FAT-PROT_), five coagulation traits for pressed cooked cheese (PCC) and soft cheese (SC) (K10/RCT_PCC_, K10/RCT_SC_, a_PCC_, a_SC_, and a2_SC_), and milk pH after adding starter for PCC (pH_0_PCC_). The accuracies of MIR predictions, assessed by the coefficient of determination (R^2^), varied between 0.54 and 0.89 depending on the CMP trait (Table 1). Milk composition was also predicted using equations that were developed in previous projects (0.44 < R^2^ < 1; Table 1). Milk proteins and fatty acids were predicted with equations that were developed in the PhénoFinlait project [12–14], whereas for minerals and citrate content we used equations that were generated by the Optimir project [15]. Lactose was predicted by a Foss equation.

**Table 1:** Means, standard deviations (SD) for cheese-making properties and milk composition traits in the genotyped population (N = 19,586), and accuracy of MIR predictions equations (R^2^_val_)

Trait	Description and unit	Mean	SD	R^2^_val_
Cheese-making properties^a^				
CY_FRESH_	100 × (g curd/g milk), in %	37.7	4.95	0.82
CY_DM_	100 × (g DM curd/g DM milk), in %	66.8	3.31	0.89
CY_FAT-PROT_	(g milk fat + g milk protein)/kg curd, in g kg^−1^	189.7	14.3	0.54
a_PCC_	Curd firmness at rennet coagulation time (RCT), in firm index (FI)	18.8	1.72	0.76
K10/RCT_PCC_	Curd organization index standardized for RCT	0.37	0.06	0.68
a_SC_	Curd firmness at RCT, in FI	18.9	1.80	0.76
a2_SC_	Curd firmness at 2 times RCT, in FI	22.8	1.41	0.69
K10/RCT_SC_	Curd organization index standardized for RCT	0.37	0.07	0.72
pH_0_PCC_	Initial value of pH	6.52	0.04	0.62
Protein composition				
PC	Protein content, in g/100 g milk	3.36	0.20	1.00
α-LA	α-lactalbumin, in g/100 g protein	4.01	0.20	0.59
β-LG	β-lactoglobulin, in g/100 g protein	12.4	1.09	0.74
αs1-CN	αs1-casein, in g/100 g protein	32.2	0.18	0.88
αs2-CN	αs2-casein, in g/100 g protein	9.73	0.19	0.82
β-CN	β-casein, in g/100 g protein	29.7	0.68	0.92
κ-CN	κ-casein, in g/100 g protein	8.74	0.24	0.80
ΣCN	Total caseins, in g/100 g protein	80.8	0.74	0.98
ΣWP	Total whey proteins, in g/100 g protein	16.9	1.15	0.54
Fatty acid composition				
FC	Fat content, in g/100 g milk	3.73	0.32	1.00
SFA	Saturated fatty acids, in g/100 g fat	70.6	3.05	1.00
MUFA	Mono-unsaturated fatty acids, in g/100 g fat	26.5	2.68	0.97
UFA	Unsaturated fatty acids, in g/100 g fat	30.0	2.93	0.98
PUFA	Poly-unsaturated fatty acids, in g/100 g fat	3.33	0.39	0.76
Σ C4-C10	Sum of C4 to C10 fatty acids, in g/100 g fat	11.6	0.71	0.95
Σ C4-C12	Sum of C4 to C12 fatty acids, in g/100 g fat	14.2	0.93	0.95
C14:0	Myristic acid, in g/100 g fat	11.1	1.05	0.94
C16:0	Palmitic acid, in g/100 g fat	28.8	2.53	0.94
C18:0	Stearic acid, in g/100 g fat	10.5	1.42	0.84
C18:1	Oleic acid, in g/100 g fat	23.2	2.59	0.96
Minerals				
Ca	Calcium, in mg/kg milk	1165	69.6	0.82
P	Phosphorous, in mg/kg milk	1014	62.5	0.75
Mg	Magnesium, in mg/kg milk	100.9	5.5	0.77
K	Potassium, in mg/kg milk	1496	69.3	0.68
Na	Sodium, in mg/kg milk	338.3	29.1	0.44
Other compounds				
Lactose	Lactose, in g/kg milk	49.3	1.4	0.92
Citrate	Citrate, in g/kg milk	0.83	0.11	0.90

^a^For pressed cooked cheese (PCC) and soft cheese (SC)

Prediction equations were applied to the original dataset, which comprised 6,670,769 milk samples originating from 410,622 Montbéliarde cows. Data from cows with at least three test-day records during the first lactation (1,442,371 test-day records from 189,817 cows) were adjusted for non-genetic effects in a mixed model with the Genekit software [16]. Herd × test-day × spectrometer, age at calving, and stage of lactation were included in this model as fixed effects, while animal genetic and permanent environmental effects were assumed to be random. Test-day data adjusted for fixed effects were then averaged over a lactation for each cow. A subset of 19,586 cows for which MIR spectra were available had also been genotyped for the purpose of genomic selection by using the BovineSNP50 (50 K, 6505 cows) or the EuroG10 K BeadChip (Illumina Inc., San Diego, 13,081 cows). Means and standard deviations of the traits for this subset are in Table 1. Using FImpute software [17], all genotypes were imputed to the 50 K-SNP level. A total of 43,801 autosomal SNPs were retained after quality control filters were applied. These filters were taken directly from the French national evaluation system [18]: individual call rate higher than 95%, SNP call rate higher than 90%, minor allele frequency (MAF) higher than 1% in at least one major French dairy cattle breed, and genotype frequencies in Hardy–Weinberg equilibrium with *P *> 10^−4^.

### Imputation to whole-genome sequences

The 50 K SNP genotypes of the 19,586 cows were then imputed to whole-genome sequences (WGS). A two-step approach was applied in order to improve the accuracy of imputed genotypes of the WGS variants [19]: from 50 to 777 K high-density (HD) SNPs using FImpute software [17], and then, from imputed HD SNPs to WGS, using Minimac software [20]. In spite of a longer computing time, Minimac was preferred over FImpute to impute on WGS because it infers allele dosages in addition to the best-guess genotypes. Compared to the best-guess genotypes, allele dosages are expected to be more correlated to true genotypes [21] and to lead to a better targeting of causative mutations in GWAS analyses [22]. Imputations from 50 K to the HD SNP level were performed using a within-breed reference set of 522 Montbéliard bulls that were genotyped with the Illumina BovineHD BeadChip (Illumina Inc., San Diego, CA) [23]. WGS variants were imputed from HD SNP genotypes using WGS variants of 2333 *Bos taurus* animals, from the 6th run of the 1000 Bull Genomes Project [21, 24]. These animals represent 51 cattle breeds and include 54 Montbéliard individuals, most of them being major ancestor bulls with a high cumulated contribution to the breed (80.6%). We applied the protocol defined by the “1000 Bull Genomes” consortium [4, 25]: (1) short reads were filtered for quality and aligned to the UMD3.1 reference sequence [4, 26], and small genomic variations (SNPs and indels) were detected using SAMtools 0.0.18 [27]; (2) raw variants were filtered to produce 26,738,438 autosomal variants as described in Boussaha et al. [26]; and (3) filtered variants were annotated with the Ensembl variant effect predictor (VEP) pipeline v81 [28] and effects of amino-acid changes were predicted using the SIFT tool [29].

**Fig. 1:**
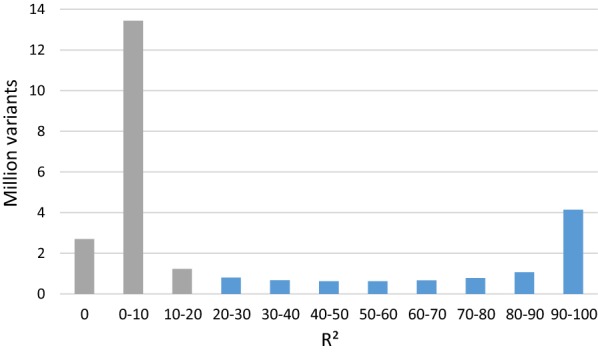
Distribution of imputation accuracies (coefficient of determination, R^2^) for 26.7 million sequence variants imputed with Minimac. Variants in blue, with R^2^ > 0.20, were retained for GWAS analyses

The precision of imputation from HD SNP to sequence was assessed using the coefficient of determination (R^2^) calculated with Minimac software [20]. In order to remove variants with low imputation accuracies, only variants with an R^2^ higher than 20% and a MAF higher than 1% were retained for further association analyses, i.e. 8,551,748 variants with a mean R^2^ of 76% (Fig. 1).

### Whole-genome sequence association analyses

We performed single-trait association analyses between all 8,551,748 variants and the 36 CMP and milk composition traits described in Table 1. All association analyses were performed using the *mlma* option of the GCTA software (version 1.24), which applies a mixed linear model that includes the variant to be tested [30]:

1 $$ {\mathbf{y}} = {\mathbf{1}}\upmu + {\mathbf{x}}{\text{b}} + {\mathbf{u}} + {\mathbf{e}}, $$

where ${\mathbf{y}}$ is the vector of pre-adjusted phenotypes, averaged per cow; $\upmu$ is the overall mean; ${\text{b}}$ is the additive fixed effect of the variant to be tested for association; ${\mathbf{x}}$ is the vector of predicted allele dosages, varying between 0 and 2; ${\mathbf{u}} \sim {\text{N}}(\mathbf{0}, {\mathbf{G}}\upsigma_{\text{u}}^{2} )$ is the vector of random polygenic effects, with $\mathbf{G}$ the genomic relationship matrix (GRM), calculated using the HD SNP genotypes [31], and $\upsigma_{\text{u}}^{2}$ is the polygenic variance, estimated based on the null model $({\mathbf{y = 1}}\upmu\,{ + }\,\varvec{ }\,{\mathbf{u + e)}}$ and then fixed while testing for the association between each variant and the trait of interest; and ${\mathbf{e}} \sim{\text{N}}(\mathbf{0}, {\mathbf{I}}\boldsymbol{\upsigma}_{\mathbf{e}}^{2} )$ is the vector of random residual effects, with $\mathbf{I}$ the identity matrix and $\upsigma_{\text{e}}^{2}$ the residual variance.

The Bonferroni correction was applied to the thresholds in order to account for multiple testing. We used a very stringent correction, which considered all 8.5 million tests as independent. Therefore, the 5% genome-wide threshold of significance corresponded to a nominal *P* value of 5.8 × 10^−9^ (− log_10_(*P*) = 8.2). When a given trait was significantly affected by multiple variants, the variants that were located less than 1 Mbp apart were grouped in the same QTL region. The bounds of QTL regions were then determined by considering the positions of variants that were included in the upper third of the peak. For each trait, the percentage of phenotypic variance explained by each QTL was calculated as follows: $\%  \sigma_{P}^{2} = 100 \left( {\frac{{2p\left( {1 - p} \right)\alpha^{2} }}{{\sigma_{P}^{2} }}} \right)$, with $\sigma_{P}^{2}$ the phenotypic variance of the trait, and $p$ and $\alpha$ are the frequency and the estimated allelic substitution effect, respectively, of the variant with the most significant effect in the QTL region.

### Co-associated gene network analysis

Co-associated genes were detected from the GWAS results using the AWM approach [5, 6]. We first constructed two $n$ × $m$ matrices with variants row-wise ($n$ = 8,551,748) and traits column-wise ($m$ = 36). The first matrix contained variants’ z-score standardized additive effects, whereas the second one contained the *P*-values associated with those effects. Among the CMP traits, CY_DM_ was selected as the key phenotype because it has the highest economic importance to the cheese-making process. The AWM was constructed following the procedure described in Ramayo-Caldas et al. [32]. SNPs were included in the analysis if their *P*-value for CY_DM_ was less than or equal to 0.001. Due to the large number of traits analyzed, we calculated correlation coefficients between SNP additive effects for different traits and then selected the set of traits correlated with CY_DM_ (|r| ≥ 0.25). Next, we explored the dependency among traits and we estimated that on average, six other phenotypes were associated with these SNPs at the same P-value (*P* ≤ 0.001). Other variants with significant effects on at least six traits were finally included in the analysis. Based on VEP annotation [33], we then selected only the SNPs that were located within or close to (within 10 kb of) genes. Among these, we retained only one variant per gene, i.e. the SNP that was associated with the largest number of traits or, in case of a tie, the variant for which the sum of P-values for the traits was the lowest.

Subsequently, to identify significant gene–gene interactions, partial correlations were computed using the PCIT algorithm developed by Reverter and Chan [34]; the algorithm was implemented in an R package designed for this purpose [35]. We visualized the gene network with Cytoscape 3.6.1 [36], with each node representing a gene and each edge representing a significant interaction. The centrality parameters of each node were assessed using the CentiScaPe 2.2 plug-in for Cytoscape [37]. For each node, we calculated the number of adjacent genes (degree parameter) and the relative node contribution (eigenvector parameter), with the latter value being higher (or lower) if the gene was connected to highly (or poorly) connected genes.

### Identification of key regulators

Potential key regulators of the gene network were identified using two approaches. First, we used the iRegulon 1.3 plug-in for Cytoscape [38] to identify transcription factors (TF) in silico; this method was based on human datasets but included orthologous regions of ten other vertebrate genomes, including *Bos taurus*. Two types of data were used to identify regulatory regions that were shared by the genes identified in the network: (1) TF binding site motifs in the cis-regulatory regions, and (2) thousands of ChIP-Seq (chromatin immunoprecipitation followed by high-throughput sequencing) datasets from the ENCODE project [39] corresponding to targets of known TF. More details are in Janky et al. [38]. We then applied an information loss-less approach [6] that explored the connectivity of all regulators in the network, including TF, miRNA, and lnRNA. As recommended by Reverter and Fortes [6], we tested trios of TF genes to find the minimal set of TF genes with maximal coverage of the network.

### Gene-set enrichment analysis

Next, we searched in the gene network for enrichment in gene ontology (GO) terms and pathways from the Kyoto Encyclopedia of Genes and Genomes (KEGG), using the ClueGO 2.5.1 plug-in for Cytoscape [40]. In order to avoid selecting GO terms that were too general (too many genes) or too specific (too few genes), we selected the 4th to 8th levels of the GO hierarchy. A gene set was considered to be enriched if the *P*-value associated with the hypergeometric test was lower than 0.05, after application of the Benjamini–Hochberg correction for multiple testing. GO terms and KEGG pathways were subsequently clustered in functional groups if the kappa statistic was higher than 0.4.

## Results

### GWAS analyses

GWAS that was carried out on 8,551,748 imputed WGS variants for the 36 CMP and milk composition traits revealed 236,332 significant variant × trait combinations (− log_10_(*P*) > 8.2), corresponding to 79,803 different variants. Due to the high maximal − log_10_(*P*) value for a large number of genomic regions (up to 560 for one of the QTL detected on chromosome 11), the number of variants with significant effects (− log_10_(*P*) > 8.2) was sometimes very large in a given region. Thus, to best target candidate variants, we selected only the variants that were located in the upper third of the peaks, as described in the Methods section. In doing so, we defined 59 QTL regions, which contained 6757 distinct variants (Table 2). In each of the QTL regions, we designated “candidate variants” as the variants that were located within the confidence intervals of the QTL and the “best candidate variant” (described in Table 2) as the variant within a gene (or its upstream/downstream regions) with the most significant effects.

**Table 2:** The 59 QTL regions identified by GWAS, the most likely candidate variant, and number of traits affected by the QTL

QTL region						Best candidate variant^a^											Number of traits^d^			
N	BTA	From (bp)	To (bp)	# variants	#genes	bp	Variant ID	Rank	R^2^	MAF	Effect	SE	− log_10_P	Gene	Functional annotation	Trait most strongly affected	CMP	Proteins	Fatty acids	Minerals
1	1	144,389,419	144,398,814	6	1	144,395,375	rs136069703	1	0.97	0.38	29.5	0.951	210.4	SLC37A1	Intron	P	3	1	1	4
2	2	5,718,384	6,567,522	111	5	5,865,070	rs136830094	1	0.91	0.22	− 0.02	0.003	15.6	INPP1	Downstream	Lactose	0	1	0	2
3	2	131,808,301	131,888,417	88	1	131,816,616	rs133677653	1	0.91	0.49	− 0.05	0.003	41.4	ALPL	Intron	κ-CN	1	3	0	1
4	3	7,442,755	8,149,715	56	5	7,935,102	rs383033753	7	0.50	0.14	− 0.65	0.096	10.7	FCGR2B	Intron	CY_DM_	2	0	1	0
5	3	15,514,034	15,928,379	401	7	15,525,599	rs110073735	1	0.99	0.03	− 2.78	0.410	10.9	EFNA1	Intron	Lactose	1	0	0	3
6	3	34,235,208	34,355,357	35	4	34,327,146	rs210558120	1	0.34	0.43	0.26	0.035	12.9	KIAA1324	Intron	a_SC_	2	0	0	0
7	4	49,033,707	49,153,995	39	2	49,033,707	rs380575157	1	0.47	0.20	0.02	0.001	30.7	CBLL1	Upstream	pH0__PCC_	2	3	0	2
8	4	75,743,094	79,803,738	59	5	77,825,429	rs385069094	1	0.57	0.01	− 2.34	0.360	10.1	GCK	Intron	Mg	0	1	0	3
9	4	92,588,016	92,966,245	227	7	92,624,543	rs379514460	1	0.34	0.03	− 29.0	4.226	11.2	FSCN3	Upstream	Ca	0	0	0	1
10	5	29,947,476	31,423,430	315	25	29,947,476	rs442522314	2	0.63	0.09	− 2.26	0.383	8.4	COX14	Downstream	Lactose	0	0	0	1
11	5	93,892,583	93,945,738	9	1	93,943,700	rs210744452	1	0.77	0.05	0.11	0.010	27.7	MGST1	Intron	FC	2	0	7	0
12	5	117,126,900	119,221,867	51	3	117,972,265	rs525880746	1	0.79	0.04	42.4	2.186	83.0	GRAMD4	Upstream	Ca	7	4	1	6
13	6	37,857,989	38,326,250	268	8	38,326,250	rs382477515	1	0.37	0.42	− 0.10	0.017	9.5	IBSP	Upstream	β-CN	0	1	0	0
14	6	46,555,489	47,082,793	89	3	46,876,802	rs110408618	1	1.00	0.17	27.8	1.189	120.5	SEL1L3	Intron	K	4	5	1	4
15	6	87,125,482	87,961,577	143	7	87,392,899	rs382350292	3	0.53	0.23	0.03	0.002	76.4	CSN3	Downstream	K10/R_SC_	8	8	0	1
16	6	108,967,622	109,145,154	91	5	109,026,822	rs208213463	1	0.93	0.47	− 8.42	0.904	19.9	GAK	Intron	Ca	2	2	0	2
17	7	958,741	1,146,962	359	4	975,515	rs383246531	1	0.93	0.37	− 1.56	0.180	17.4	47695^b^	Intron	Lactose	0	0	0	2
18	7	41,576,519	46,646,915	399	23	46,452,656	rs209828204	6	0.87	0.07	1.36	0.127	26.0	FSTL4	Downstream	a_PCC_	2	2	0	1
19	9	102,731,669	103,130,410	207	3	102,885,120	rs134445867	15	0.90	0.03	− 0.14	0.023	9.3	MPC1	Intron	C4_C10	0	0	1	0
20	10	1,984,741	2,326,212	86	1	2,096,282	rs385793060	67	0.99	0.35	− 2.04	0.380	7.1	47622^b^	Upstream	Na	0	0	0	1
21	10	48,359,318	50,266,445	213	1	49,459,919	rs109896326	1	1.00	0.14	0.30	0.046	9.8	RORA	Intron	UNSAT	0	0	4	0
22	10	99,714,774	99,843,583	20	0	99,714,774	rs440530756	1	0.22	0.02	− 0.18	0.028	9.8	–	Intergenic	PUNSAT	0	0	2	0
23	11	9,023,594	9,684,851	11	3	9,684,851	rs384459785	8	0.62	0.07	− 2.82	0.385	12.6	POLE4	Intron	CY_FAT-PROT_	3	0	3	0
24	11	14,152,677	15,493,112	132	6	14,284,886	rs384594145	48	0.90	0.10	− 0.01	0.001	18.7	XDH	Upstream	pH_0___PCC_	1	0	0	1
25	11	86,907,041	86,916,295	9	1	86,912,990	rs481567394	1	0.21	0.05	0.02	0.003	16.7	ATP6V1C2	Intron	pH_0___PCC_	1	0	0	0
26	11	103,273,963	103,322,890	214	4	103,301,982	rs109907194	48	0.89	0.46	− 1.09	0.017	559.7	PAEP	Intron	ΣWP	8	7	2	3
27	12	68,616,690	77,578,414	337	7	70,162,028	rs721489054	22	0.83	0.17	0.22	0.019	29.0	ABCC4	Intron	C14:0	0	0	7	2
28	13	20,094,707	22,437,171	291	4	21,053,894	rs378591536	7	0.34	0.07	− 0.24	0.034	12.1	23216^b^	Intron	C14:0	0	0	1	0
29	13	45,394,264	48,611,254	75	4	46,734,011	rs379821485	18	0.76	0.15	− 0.01	0.001	10.6	RF0026	Downstream	pH_0___PCC_	1	0	1	0
30	13	52,289,279	55,114,121	80	9	54,938,610	rs110422533	2	1.00	0.39	− 6.60	1.007	10.3	GID8	Synonymous	K	0	0	2	1
31	13	64,648,620	64,870,118	19	3	64,812,464	rs43717459	1	0.33	0.10	− 0.24	0.027	18.9	ACSS2	Intron	C4–C10	0	0	1	0
32	13	79,225,285	79,326,265	6	1	79,326,265	rs109862148	3	0.38	0.07	− 0.04	0.007	8.9	FAM65C	Downstream	αs2-CN	0	1	0	0
33	14	1,622,956	1,881,116	27	9	1,629,753	rs109035586	1	0.36	0.33	0.17	0.007	122.7	GPT	Upstream	FC	6	6	10	4
34	15	39,854,757	40,283,508	140	2	39,885,845	rs134953698	2	0.79	0.18	− 0.04	0.005	10.4	ARNTL	Intron	PUNSAT	0	0	1	0
35	15	52,993,384	54,612,060	341	10	53,943,342	rs381948106	1	0.47	0.04	17.5	2.747	9.7	RAB6A	Intron	P	0	0	2	2
36	16	1,607,369	3,050,452	10	0	1,609,129	rs42450079	1	0.54	0.28	− 0.04	0.005	17.0	–	Intergenic	κ-CN	2	6	0	0
37	16	60,539,357	63,891,341	178	3	60,646,127	rs137615589	72	0.75	0.14	0.02	0.004	9.8	38238^b^	Upstream	a_SC_	2	1	0	0
38	16	67,700,538	67,811,269	167	2	67,758,163	rs42465711	1	1.00	0.4	0.31	0.044	11.9	SWT1	Intron	CYDM	2	1	2	1
39	16	70,170,086	71,493,899	59	1	71,432,479	rs109766366	5	0.79	0.43	− 0.23	0.032	12.3	PROX1	Intron	UNSAT	0	0	4	2
40	17	29,348,215	30,211,790	203	9	29,938,428	rs207509104	21	0.55	0.36	0.10	0.015	10.3	LARP1B	Synonymous	C4–C10	0	0	1	0
41	17	52,753,338	53,240,467	283	6	53,072,959	rs448501071	5	1.00	0.06	− 0.32	0.019	62.4	BRI3BP	Intron	C4–C10	2	3	2	1
42	18	10,566,605	11,091,131	68	5	11,002,789	rs41867427	2	0.61	0.06	14.3	2.071	11.3	CRISPLD2	Intron	a_SC_	2	0	0	1
43	19	51,304,834	51,538,272	67	2	51,383,847	rs136067046	1	0.83	0.32	0.20	0.013	49.4	FASN	Upstream	C14:0	0	0	9	0
44	19	55,229,384	57,240,571	267	10	57,151,350	rs42848485	1	0.63	0.46	9.07	1.079	16.4	FADS6	Intron	Ca	2	1	1	1
45	19	60,407,923	62,177,206	142	0	61,135,270	rs41923848	1	0.91	0.13	− 2.28	0.210	26.6	–	Intergenic	Lactose	0	3	0	3
46	20	58,245,970	58,457,768	87	1	58,446,058	rs137085630	22	0.99	0.06	− 1.01	0.036	176.5	ANKH	Intron	Citrate	5	4	2	5
47	21	40,120,343	44,138,058	63	2	41,638,428	rs137153434	25	0.21	0.12	0.07	0.009	12.7	G2E3	Upstream	PC	3	1	0	1
48	22	32,877,755	33,466,544	46	2	32,877,755	rs208141216	10	0.30	0.08	0.08	0.007	25.7	FAM19A4	Intron	PC	8	1	1	2
49	22	55,186,094	55,273,619	72	1	55,254,221	rs43597796	1	0.93	0.49	0.00	0.001	12.6	ATP2B2	Intron	pH_0___PCC_	1	1	0	0
50	22	61,257,725	61,312,492	18	1	61,284,069	rs109001472	1	1.00	0.47	− 0.09	0.008	28.3	KLF15	Intron	C4–C10	0	0	2	0
51	24	50,420,365	50,550,020	114	2	50,465,348	rs383068825	4	0.53	0.34	− 10.7	1.282	16.0	SKA1	Downstream	K	1	1	0	2
52	24	58,744,952	58,825,217	23	3	58,817,202	rs208779762	1	0.93	0.43	− 6.57	0.807	15.4	LMAN1	Upstream	P	1	1	0	1
53	25	2,994,081	3,261,509	28	3	3,241,838	rs137696417	16	0.61	0.18	− 0.03	0.007	6.5	ADCY9	Downstream	κ-CN	0	1	0	0
54	25	25,642,563	29,605,418	82	11	26,498,356	rs137150057	1	1.00	0.34	− 0.11	0.020	11.3	FAM57B	5′UTR	C18:0	1	1	6	0
55	26	20,727,700	21,427,109	61	4	21149234	rs136334180	11	1.00	0.31	0.39	0.035	27.6	SCD	Upstream	UNSAT	0	0	11	0
56	26	32,773,808	33,925,908	95	2	33,233,277	rs385554497	36	0.27	0.02	1.55	0.270	8.1	ACSL5	intron	a_PCC_	1	0	0	0
57	27	36,165,492	36,235,730	11	1	36,212,352	rs208675276	1	0.54	0.41	0.65	0.040	60.4	GPAT4^c^	5′ UTR	C16:0	3	0	7	1
58	28	6,008,464	7,038,810	141	3	6,027,037	rs382911338	1	0.37	0.18	− 0.01	0.001	17.1	PCNX2	Intron	pH_0___PCC_	1	2	0	1
59	29	9,253,006	9,622,389	145	3	9,343,362	rs133715120	6	0.61	0.33	− 2.76	0.264	24.9	EED	Intron	Lactose	0	3	1	4

^a^When a gene was present in the confidence interval of the QTL, the best candidate variant was the genic variant with the most significant effects (intergenic variants were discarded)

^b^47695, 47622, 23216, and 38238 for ENSBTAG00000047695, ENSBTAG00000047622, ENSBTAG00000023216, and ENSBTAG00000038238, respectively

^c^Also named *AGPAT6*

^d^Number of milk cheese-making (CMP), protein, fatty acid, and mineral composition traits with significant effects

**Fig. 2:**
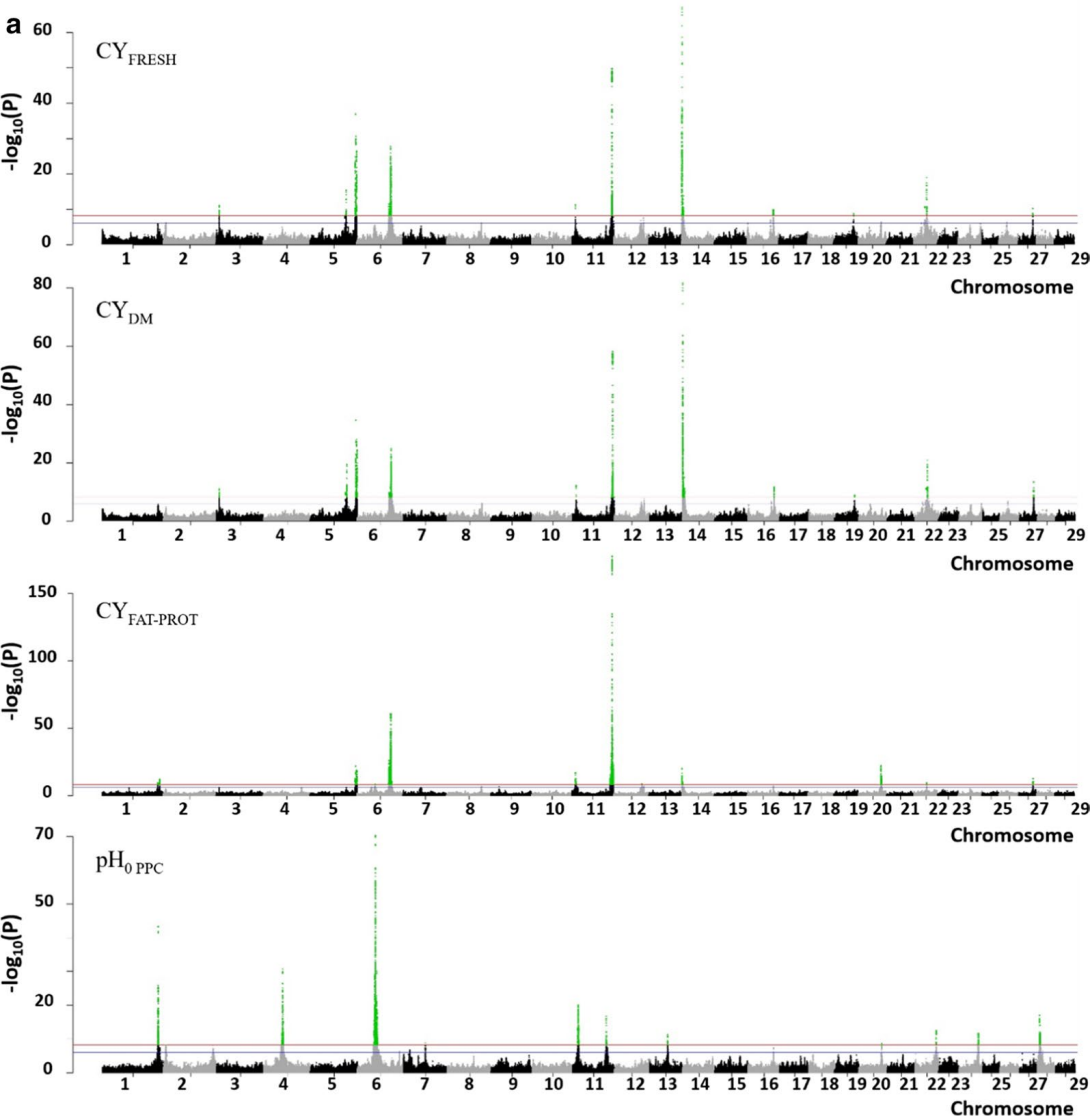

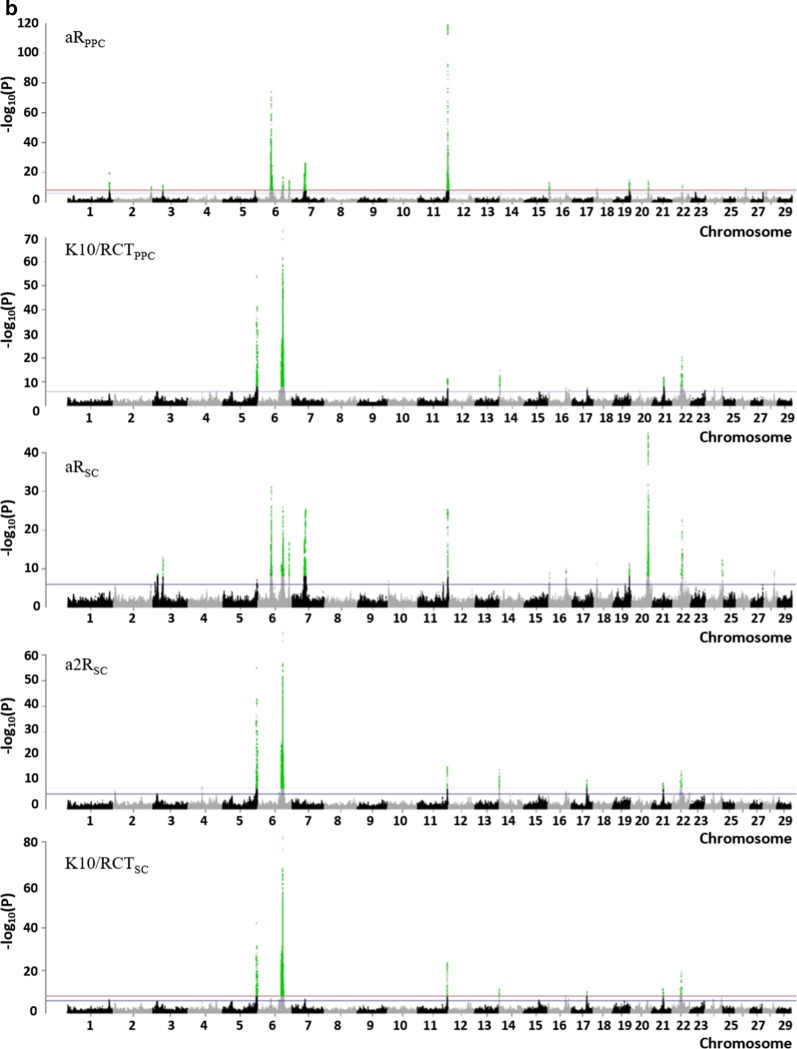
−log_10_(P) values plotted against the position of variants on *Bos taurus* autosomes for cheese-making traits. **a** Cheese yields (CY) and pH_0 PPC_, **b** coagulation traits

These QTL regions varied in size (from 9.2 kbp to 8.9 Mbp) and contained from 6 to 401 variants; they were distributed on all *Bos taurus* autosomes (BTA) with the exception of BTA8 and 23 (Fig. 2 and [see Additional file 1: Figure S1]). In almost all the QTL regions (56), we identified variants that were located in one or more candidate genes. Around 60% (i.e. 4312 of 7393) of the variants detected in the QTL regions were located within or in the upstream/downstream region of 264 genes [see Additional file 2: Table S1]. Fifty-one of these variants were predicted to be responsible for an amino-acid change in the protein, whereas most of them (2972) were located in introns (Table 3).

**Table 3:** Functional annotations of variants included in the 59 QTL regions

Functional annotation	Number of variants	%
Intergenic	3081	41.7
Intronic	2972	40.2
Upstream	604	8.2
Downstream	584	7.9
3′ UTR	26	0.35
5′ UTR	10	0.14
Synonymous	65	0.88
Missense	51	0.69
Total	7393	100

We found the most significant effects around 103.3 Mbp on BTA11 (− log_10_(*P*) = 560), 144.4 Mbp on BTA1 (− log_10_(*P*) = 210), 58.4 Mbp on BTA20 (− log_10_(*P*) = 177), 1.6 Mbp on BTA14 (− log_10_(*P*) = 123), and 46.9 Mbp on BTA6 (− log_10_(*P*) = 120). In each of these five QTL, we identified variants that were located in candidate genes, which were, respectively, *PAEP*, *SLC37A1*, *ANKH*, *GPT*, and *SEL1L3*. All the variants were located in introns of the genes, with the exception of the best candidate variant of the *GPT* gene, which was found in the upstream region. Four other QTL had more moderate but nevertheless strong effects (− log_10_(*P*) between 60 and 83), on BTA5 (118 Mbp), BTA6 (87.4 Mbp), BTA17 (53.1 Mbp), and BTA27 (36.2 Mbp), with the best candidate variants located in *GRAMD4* (upstream region), *CSN3* (downstream region), *BRI3BP* (upstream region), and *GPAT4* (3’UTR region), respectively. We also found candidate variants (− log_10_(*P*) between 25 and 50) in 11 other candidate genes, on BTA2 (*ALPL*), BTA4 (*CBLL1*), BTA5 (*MGST1*), BTA7 (*FSTL4*), BTA12 (*ABCC4*), BTA19 (*FASN*), BTA22 (*FAM19A4* and *KLF15*), BTA25 (*FAM57B*), BTA26 (*SCD*), and BTA29 (*EED*). Finally, many other variants were identified in various genomic regions that had more moderate but significant effects after application of the Bonferroni correction (− log_10_(*P*) > 8.2); most of these were located in genes. All the QTL regions are described in detail in Table 2.

On average, each QTL had significant effects on about six traits. Only 13 QTL affected a single trait, while the other 46 QTL had pleiotropic effects on two to 26 traits. The QTL that affected the largest number of traits was located at about 1.6 Mbp on BTA14. For most traits, including FC, the variant with the strongest effect was not the well-known K232A polymorphism in the *DGAT1* gene [see Additional file 3: Table S2]. More than half of the QTL (33), and in particular those with the most significant effects, had effects on CMP traits. Almost all of the QTL with significant effects on CMP traits presented significant pleiotropic effects on milk composition traits, as well. In contrast, the remaining 26 QTL affected milk composition (protein, fatty acid, mineral, citrate, or lactose content) but not CMP. Among traits, we observed large differences in both the number of QTL detected (ranging from 6 to 19) and in the total percentage of phenotypic variance (ranging from 4.7 to 62.4%) that was explained by the detected QTLs, and simply estimated by the sum of percentages per QTL. Overall, the larger the number of detected QTL for a given trait, the lower the percentage of phenotypic variance that was explained by each. For example, in our study, the most polygenic trait, a_SC_, was influenced by 19 QTL, each of which explained only 0.2 to 1.9% of the phenotypic variance. In contrast, we detected only six QTL for ΣWP but the QTL with the most important effect explained 56% of the phenotypic variance of this trait. As expected, the most heritable traits were those that presented the highest values of the total phenotypic variance explained by the QTL. The trait for which the largest amount of total phenotypic variance was explained by the QTL was β-LG (62%), which was also the most heritable trait analyzed in our study. For CMP traits, which are moderately heritable, from 12% (curd firmness) to 30% (curd firming time) of the phenotypic variability was explained by the QTL (i.e. from 27 to 65% of the genetic variance). Cheese yields presented intermediate results, as the detected QTL captured about 20% of their phenotypic variance, i.e. about 50% of their genetic variance. For CMP traits, the QTL that contributed the most were those detected in the regions of the *PAEP*, *casein*, and *DGAT1* genes. However, other QTL regions on BTA5, 6, 16, 20, and 22 also generated noteworthy contributions. For protein composition traits, the highest-contributing QTL region was the *PAEP* gene region (up to 59% for β-LG). The region of the *casein* genes had a more moderate contribution (0.7–5.6%, depending on the trait), while the lesser-known QTL detected on BTA20 (at about 58 Mbp) explained 18, 9, and 7% of the phenotypic variance of α-LA, αs1-CN, and κ-CN, respectively. For fatty acid content, the QTL that we detected explained a much smaller part of the phenotypic variability. The top-contributing QTL were the *DGAT1* gene region on BTA14 (12% for FC), *FASN* on BTA19 (1.5% for C14:0), *GPAT4* on BTA27 (3.2% for C16:0), and *SCD* on BTA26 (2% for C18:1). In contrast to fatty acids but similarly to proteins, a relatively large part of the phenotypic variance in mineral content was explained by QTL that were located in the region of the *SLC37A1* gene (3, 5, and 10% for Mg, K, and P, respectively) and the *ANKH* gene (20% for Mg). Two other regions influenced mineral content to a lesser extent: those at 117 Mbp on BTA5 (*GRAMD4*) and at 46 Mbp on BTA6 (*SEL1L3*).

### Gene network

Using the AWM procedure, we reduced the set of 8.5 million variants tested in the GWAS to a set of 38,858 variants that had the most significant effects (*P*-value ≤ 0.001) on the key phenotype (CY_DM_). Seven CMP (CY_FRESH_, CY_FAT-PROT_, and the five coagulation traits) and eight milk composition traits (PC, FC, UNSAT, PUNSAT, C18:1, Ca, Mg, and P) were correlated with CY_DM_ (r ≥ 0.25). On average, each of the 38,858 variants had significant effects (P-value ≤ 0.001) on six of the correlated traits. We also retained 2322 additional variants that had significant effects on at least six of the correlated phenotypes. Thus, the final dataset included 41,180 variants, which had significant effects on CY_DM_ or on at least six correlated traits. Of these 41,180 variants, 15,330 were located in 736 genes (± 10 kb); the PCIT approach subsequently revealed 59,168 significant interactions among these genes. Thus, by merging the AWM and the PCIT approaches, the GWAS results on milk CMP and composition traits could be interpreted as a gene network of 736 nodes and 59,168 edges. The list of the 736 genes selected by AWM is in [see Additional file 4: Table S3].

For most of the traits, correlation coefficients from the z-score additive effects of the 736 variants retained by the AWM procedure were close to the correlation coefficients obtained from pedigree for the 16 phenotypes (Table 4). This suggested that the additive effects of the variants retained in the AWM analysis explained a large and representative part of the genetic relationships among the traits.

**Table 4:** Genomic correlations calculated using additive effects of the 736 SNPs selected by the AWM (above the diagonal) and genetic correlations estimated from pedigree or taken from Sanchez et al. [3] (below the diagonal)

	CY_FRESH_	CY_DM_	CY_FAT-PROT_	K10/RCT_PCC_	a_PCC_	K10/RCT_SC_	a_SC_	a2_SC_	PC	FC	C18:1	UFA	PUFA	Ca	Mg	P
CY_FRESH_		1.00	− 0.87	− 0.84	0.25	− 0.84	0.52	0.85	0.84	0.96	− 0.82	− 0.78	− 0.80	0.70	0.52	0.83
CY_DM_	0.97		− 0.88	− 0.85	0.27	− 0.85	0.55	0.86	0.85	0.95	− 0.80	− 0.76	− 0.78	0.71	0.55	0.84
CY_FAT-PROT_	− 0.82	− 0.84		0.79	− 0.61	0.84	− 0.73	− 0.79	− 0.72	− 0.72	0.57	0.51	0.58	− 0.55	− 0.54	− 0.75
K10/RCT_PCC_	− 0.72	− 0.73	0.65		− 0.31	0.99	− 0.69	− 0.99	− 0.98	− 0.74	0.57	0.52	0.44	− 0.80	− 0.75	− 0.86
a_PCC_	0.77	0.78	− 0.65	− 0.76		− 0.41	0.79	0.29	0.20	0.01	0.08	0.14	0.02	− 0.12	0.29	0.35
K10/RCT_SC_	− 0.74	− 0.76	0.71	0.80	− 0.78		− 0.75	− 0.98	− 0.95	− 0.71	0.53	0.47	0.41	− 0.75	− 0.76	− 0.87
a_SC_	0.76	0.78	− 0.67	− 0.73	0.76	− 0.77		0.63	0.64	0.32	− 0.19	− 0.14	− 0.15	0.36	0.72	0.62
a2_SC_	0.72	0.75	− 0.64	− 0.72	0.77	− 0.77	0.74		0.97	0.75	− 0.59	− 0.53	− 0.45	0.79	0.68	0.87
PC	0.74	0.75	− 0.52	− 0.80	0.94	− 0.81	0.91	0.89		0.77	− 0.59	− 0.55	− 0.46	0.84	0.76	0.86
FC	0.91	0.87	− 0.57	− 0.53	0.55	− 0.47	0.51	0.48	0.60		− 0.89	− 0.87	− 0.87	0.69	0.41	0.75
C18:1	− 0.38	− 0.45	0.22	0.20	− 0.22	0.13	− 0.18	− 0.17	− 0.23	− 0.57		0.99	0.93	− 0.52	− 0.19	− 0.56
UFA	− 0.34	− 0.40	0.17	0.13	− 0.14	0.05	− 0.10	− 0.09	− 0.21	− 0.55	0.47		0.93	− 0.50	− 0.16	− 0.51
PUFA	− 0.47	− 0.42	0.30	0.00	− 0.02	− 0.04	0.01	0.04	− 0.29	− 0.59	0.71	0.74		− 0.42	− 0.13	− 0.50
Ca	0.41	0.40	− 0.25	− 0.46	0.45	− 0.37	0.39	0.42	0.41	0.26	− 0.30	− 0.30	− 0.35		0.76	0.71
Mg	0.54	0.58	− 0.44	− 0.58	0.59	− 0.58	0.54	0.54	0.40	0.20	− 0.25	− 0.25	− 0.05	0.60		0.70
P	0.40	0.41	− 0.31	− 0.54	0.50	− 0.53	0.42	0.40	0.40	0.29	− 0.31	− 0.30	− 0.39	0.34	0.58	

Among the 736 genes, 86 were located within QTL regions that had been highlighted by the GWAS analysis with a most-stringent threshold; these included the best candidate genes for 25 QTL. The remaining 650 genes were unique to the AWM analysis and had not been detected by GWAS. In contrast, 178 genes located within the confidence intervals of QTL detected with GWAS were not found in AWM analyses.

**Fig. 3:**
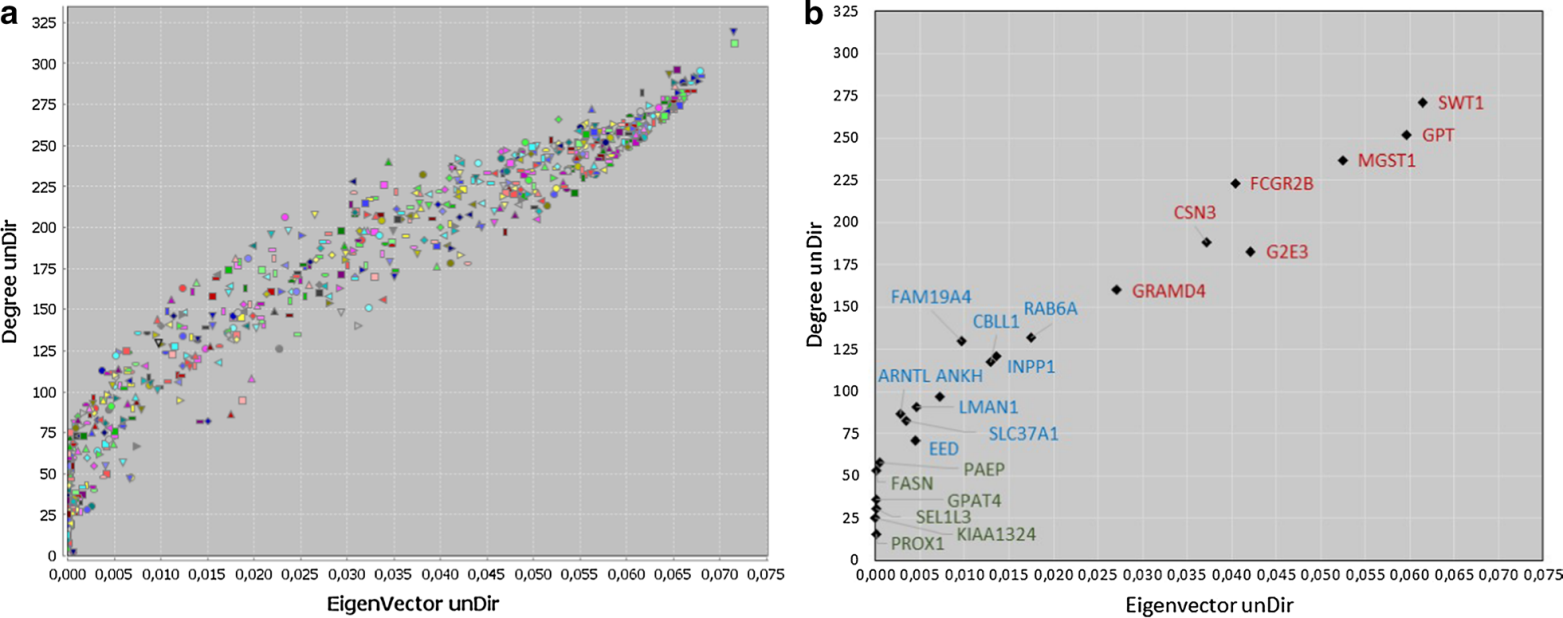
Centiscape scatter plot view: number of adjacent genes (degree) and the relative node contribution (eigenvector) **a** for the 736 genes of the gene network and **b** for the best candidate genes

For each node of the gene network, we calculated the number of adjacent genes and the relative node contribution. Figure 3 lists the values of these parameters for the nodes of the gene network that were also best candidate genes in the GWAS analyses. This revealed genes that were highly connected with other genes in the network (*SWT1*, *GPT*, *MGST1*, *FCGR2B*, *CSN3*, *G2E3*, and *GRAMD4*), genes that were moderately connected (*RAB6A*, *FAM19A4*, *INPP1*, *CBLL1*, *ANKH*, *LMAN1*, *ARNTL*, *SLC37A1*, and *EED*), and genes that were poorly connected (*PAEP*, *FASN*, *GPAT4*, *SEL1L3*, *KIAA1324*, and *PROX1*).

### In silico functional analyses

Key regulators in the gene network were identified in silico using two approaches. From the analyses of binding site motifs and ChIP-Seq datasets, first we identified eight TF that presented a significant normalized enrichment score (NES). Each of these TF targeted from 136 to 261 genes in the gene network (Table 5), and all eight together targeted more than half of the network genes (416). Using an information loss-less approach, we then identified among the 736 genes the trios of regulators (TF, miRNA, and lnRNA) that had the best coverage of the whole gene network, i.e. trios that demonstrated the largest number of interactions with genes of the network with the least amount of overlap. With this second approach, we found 61 regulators, each with two to 276 significant interactions with genes of the network. The trios that covered the largest number of genes were *ASXL3*—*HIC2*—*RNF2* and *HIC2*—*ZPFM2*—*bta*-*mir*-*200c*. These two trios interacted with the majority of the genes of the network, i.e. 529 and 528 genes, respectively.

**Table 5:** Transcription factors (TFs) identified as key regulators of milk cheese-making and composition traits from both binding-site motifs and ChIP-Seq datasets, which presented significant normalized enrichment scores (NES)

TF	NES	Number of binding site motifs	Number of ChIP-Seq datasets	Number of target genes	Chromosome	Gene start (bp)	Gene end (bp)	Gene description
HSPA1L	4.90	5	1	261	23	27,334,344	27,338,328	Heat shock 70 kDa protein 1-like
SMAD5	4.63	4	2	253	7	49,155,483	49,217,780	SMAD family member 5
HNF1B	4.56	3	5	242	19	14,287,673	14,349,579	HNF1 homeobox B
SMAP2	4.30	7	1	236	3	106,311,859	106,358,978	Small ArfGAP2
TFAP2A	4.29	3	1	233	23	45,480,546	45,499,034	Transcription factor AP-2 alpha
BCL11A	4.25	5	1	195	11	43,071,977	43,174,031	B Cell CLL/Lymphoma 11A
SMAD3	4.02	3	1	170	10	13,958,174	13,980,371	SMAD family member 3
RXRA	3.49	2	1	136	11	105,990,344	106,015,000	Retinoid X receptor alpha

**Fig. 4:**
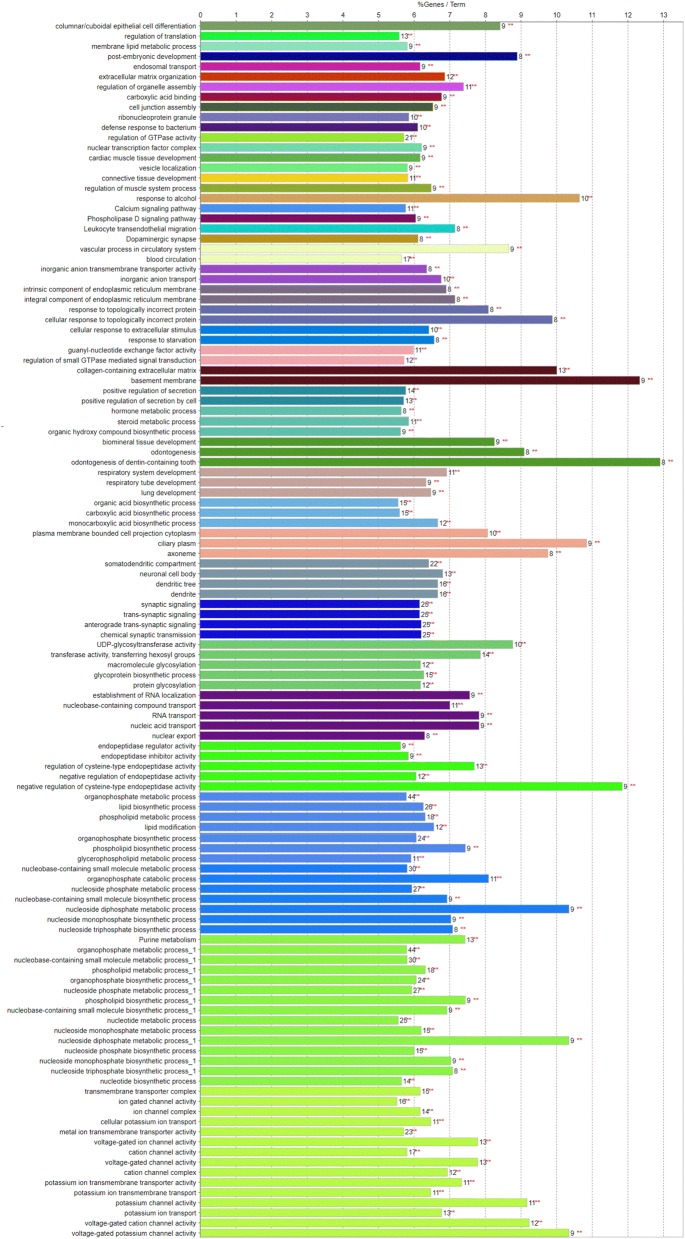
Description of the five KEGG pathways and 105 GO terms that were significantly enriched among genes of the network and which clustered in 44 functional groups

Genes of the network were found to be enriched in five KEGG pathways and 115 GO terms (corrected *P*-value between 2.10^−17^ and 2.10^−4^), which clustered into 44 functional groups (Fig. 4 and [see Additional file 5: Table S4]). The largest group comprised 15 GO terms; it contained 31 genes of the gene network and was related to the metabolic processes associated with potassium transport. The next three groups, with 28 GO terms and one KEGG pathway all related to phosphate and phospholipid metabolism, contained 66 genes of the network. Among these, there were many of the genes that had been highlighted by GWAS as having the most significant effects on milk CMP and composition traits: *CSN1S1*, *DGAT1*, *FASN*, *GPAT4*, *INPP1*, *PPARA*, *PROX1*, and *SCD*. Other groups, (for details [see Additional file 5: Table S4]), had a functional relationship with milk composition through endopeptidase activity (16 genes, including *CSN2* and *GRAMD4*), protein glycosylation (19 genes), carboxylic acid biosynthesis (24 genes including *FASN*, *PAEP*, *PPARA*, *PROX1*, and *SCD*), inorganic anion transport (10 genes including *ANKH* and *SLC37A1*), and Ca- (11 genes) and phospholipase- (9 genes) signaling pathways.

## Discussion

### GWAS and gene network analyses are complementary

The GWAS approach used here—performed on whole-genome sequences from a large number of animals for complex cheese-making traits as well as fine-scale milk composition traits—led to the identification of 59 QTL regions. In order to limit the detection of false positives, we retained only the QTL that still demonstrated significant effects after applying the Bonferroni correction (*P*-value < 5.8 × 10^−9^) and therefore those that presented the strongest effects overall. The downside of this approach was that all the QTL in our analysis explained, on average, less than 50% of the total genetic variation of each trait (i.e. 20% of the phenotypic variance), and this value was probably overestimated. Indeed, when the true effect is small or when the P-value threshold is very low, the detection power is limited and a significant effect may be overestimated, leading to an overestimation of SNP variance. Some QTL were identified with very good resolution (narrow peaks), such as the 12 QTL for which only one candidate gene was identified within the confidence interval. Other QTL regions were larger and more gene-rich (up to 25 genes within the confidence interval), and identification of the best candidate gene was not straightforward. To address these two shortcomings—specifically, to capture the missing genetic variability and to better identify functional candidate genes within QTL regions—we carried out additional analyses, which complemented our GWAS results. The AWM-PCIT approach enabled us to identify a more comprehensive gene network of 736 genes from lower significant GWAS results (*P*-value < 0.001) by taking co-associations between traits into account. When we used the additive effects of variants that were located in these genes to calculate correlations between traits, the values obtained were similar to the genetic correlations we calculated from pedigree [3], suggesting that the gene network adequately explained the genetic relationships between traits. Finally, in silico functional analyses of genes of the network helped us to identify metabolic pathways and key regulators with functional links to milk cheese-making and composition traits. This last step, in addition to establishing functional links between the gene network and the analyzed traits, enabled us to identify candidate genes in some QTL regions. Therefore, by combining the results obtained through these different approaches, we are able to propose candidate genes for the main QTL regions, and for each, the best candidate for the causative variant, or at least, a variant in high LD with the causative variant.

### Functional candidate genes

As expected, we confirmed the strong effects of the cluster of casein genes and the *PAEP* gene regions on protein composition as well as milk CMP. The QTL detected in the casein genes region explained up to 20% $\upsigma_{\text{P}}^{2}$ of the curd-firming time while the *PAEP* gene region explained up to 8.5% $\upsigma_{\text{P}}^{2}$ of cheese yields. The best candidate gene variants, i.e. variants with the most significant effects on traits, were located in the downstream region of the *CSN3* gene, which encodes κ-CN (at 87,392,899 bp on BTA6), and in an intronic region of the *PAEP* gene, which encodes β-LG (at 103,301,982 pb on BTA11). The missense variants that are respectively responsible for the κ-CN [41] and β-LG [42] A/B polymorphisms had much weaker effects: they were ranked 100th and 56th, respectively, among the variants. The region of the *DGAT1* gene on BTA14 had also large effects on milk composition (12% $\upsigma_{\text{P}}^{2}$ for FC) and on CMP (6.4% $\upsigma_{\text{P}}^{2}$ for CY_DM_). In spite of its low MAF in Montbéliarde cows (0.015), the *K232A DGAT1* mutation [43] was the top-ranked variant for traits that were linked with some protein and phosphorous contents, and coagulation traits (1st for PC, α-LA, β-CN, and P; 2nd for a2_SC_, K10/RCT_SC_, and K10/RCT_PCC_) and it was one of the 736 variants retained by the AWM. However, in this gene-rich region, the *GPT* gene, which we found to be highly connected, i.e. presenting significant gene–gene interactions with many other genes of the AWM gene network, appeared to be also a good candidate for FC, CY_DM_, CY_FRESH_, and fatty acid composition. The best candidate variant, located in the upstream region of *GPT* (*glutamic*-*pyruvic transaminase*) at 1,629,753 bp (rs109035586), was ranked 1st for 12 traits, including FC, cheese yields, fatty acid composition, αS1-CN, and CITRATE. Interestingly, two polymorphisms in the *GPT* gene, including a missense variant that is located very close to the best candidate variant detected in our study (1,629,600 bp), were also recently found to be associated with fat percentage in a concordance analysis carried out on imputed whole-genome sequences of Holstein bulls [44]. This variant was also highly significant in our study but was ranked 44th among variants with significant effects on FC.

In addition to the three well-known QTL regions described above, we also found evidence that other genomic regions have highly significant effects on the traits analyzed; specifically, our analysis highlighted the *SLC37A1*, *ALPL*, *MGST1*, *SEL1L3*, *FASN*, *ANKH*, *BRI3BP*, *SCD*, and *GPAT4* genes, which we had also previously detected in a sequence-based GWAS on milk protein and fatty acid composition [45, 46]. We confirm here their effects on milk composition and note their effects on CMP. As previously found, the *MGST1*, *FASN*, *SCD*, and *GPAT4* genes mainly affected fatty acids whereas the *SLC37A1*, *ALPL*, *SEL1L3*, *BRI3BP*, and *ANKH* genes had effects mainly on proteins and minerals. As a consequence, and in accordance with genetic correlations that we had previously estimated from this dataset [3], the former set of genes exclusively influenced cheese yields whereas the latter set had greater effects on coagulation traits. Strong effects of *ALPL*, *ANKH,* and *SEL1L3,* which we had previously identified for protein composition [45], were confirmed for milk composition and CMP. In each of these regions, the current analysis reduced the size of the confidence intervals of the QTL and, in six of them, only one gene was found that encoded a known protein (*SLC37A1*, *ALPL*, *MGST1*, *SEL1L3*, *ANKH*, and *GPAT4*).

On BTA17, we found two QTL regions associated with de novo milk fatty acid synthesis, i.e. synthesis within the mammary epithelial cells of fatty acids C4:0 to C10:0. The first was within the *LARP1B* (*La ribonucleoprotein domain family member 1B*) gene, for which the best candidate was a synonymous variant located at 29,938,428 bp. This result corroborates the discovery of Duchemin et al. [47], who identified *LARP1B* as a causative gene for de novo synthesis of milk fatty acids through the imputation of sequence variants in this region. These authors noted a splice-region variant at 29,940,555 bp, which was close to the variant that we detected here. However, in spite of its high MAF (0.40), we excluded this variant because it was not significant in our study (*P*-value = 10^−4^ vs. 5.10^−11^ for the variant located at 29,938,428 bp). This region had limited effects in our study and affected only short FA traits. Instead, further along the same chromosome, we identified another region with much more significant effects on de novo fatty acid synthesis that also affected CMP and protein and mineral composition. The best candidate gene for this region was *BRI3BP* (*BRI3 binding protein*), with the most significant variant located at 53,072,959 bp in an intron of *BRI3BP*. This variant had been previously highlighted for its effects on FA composition in an independent population [48] and, in another study, we recently confirmed its effects on both CMP and milk composition traits [46]. Thus, it is a serious candidate for the causative variant behind the strong effects that we observed in the region. Although the *BRI3BP* gene was not an obvious functional candidate, it has been also described as affecting de novo fatty acid synthesis in a recent GWAS performed on imputed sequence variants in this region [49]. The most significant variant found by the authors of this study was also intronic (53,078,216 bp) but that particular variant was ranked 31^st^ among variants with significant effects on C4–C10.

Finally, we identified other candidate genes that contained variants with non-negligible effects on milk composition and CMP traits. Among these, both GWAS and AWM analyses highlighted *FCGR2B*, *KIAA1324*, *CBLL1*, *GRAMD4*, *ARNTL*, *RAB6A*, *ENSBTAG00000038238*, *SWT1*, *G2E3*, *FAM19A4*, *LMAN1*, and *EED*. The *FCGRB2*, *KIAA1324*, *G2E3*, *LMAN1*, and *EED* genes have been previously identified as candidate genes for milk yield or milk composition [50–54], whereas the functional link between the other genes and bovine milk composition and cheese-making traits remains to be discovered.

### Co-association gene network

The *SLC37A1* (*solute carrier family 37 member 1, a phosphorous antiporter*) and *ANKH* (*inorganic pyrophosphate transport regulator*) genes, which encode transmembrane proteins involved in ion transport, both play a role in the inorganic anion transport that was revealed by the GO analysis. Thus, these genes are good candidates for having an effect on CMP and milk composition, with the strongest effects obtained for phosphorous (about 11% $\upsigma_{\text{P}}^{2}$) and citrate (about 32% $\upsigma_{\text{P}}^{2}$) contents, respectively. For each of these genes, we propose here an intronic candidate variant, located at 58,446,058 bp for *ANKH* and at 144,395,375 bp for *SLC37A1*. Very close to but distinct from those identified in previous studies [45, 53, 55], this variant is more significant in spite of a slightly lower imputation accuracy.

A set of genes, including those detected previously (*DGAT1*, *FASN*, *GPAT4*, *CSN1S1*, *PAEP*, and *SCD*) and those noted here for the first time (*INPP1*, *PPARA*, *PROX1*), appeared to play a role in phosphate and phospholipid metabolism as well as in the biosynthesis of carboxylic acids, which are fatty acid precursors. *PROX1* (*prospero homeobox 1*) and *PPARA* (*peroxisome proliferator activated receptor alpha*) encode transcription factors; the former interacted with only 16 genes while the latter interacted with 128 genes within the network, including with *FASN*, *SCD*, *GPAT4*, and *DGAT1*. *PPARA* belongs to a superfamily of hormone receptors (*PPAR*) that regulate the transcription of genes involved in different lipid metabolism pathways [56]. *FASN* (*fatty acid synthase*) and *SCD* (*stearoyl*-*coenzyme A desaturase 1)* encode key enzymes in de novo fatty acid synthesis and fatty acid desaturation, respectively, and *GPAT4* (*glycerol*-*3*-*phosphate acyltransferase 4*) is paralogous to *DGAT1* (*diacylglycerol O*-*acyltransferase 1*), with the two genes occupying adjacent nodes of the mammary triglyceride synthesis chain [57]. In addition to their effects on protein composition, the *PAEP* and *CSN1S1* genes, which encode milk β-LG and αs1-CN proteins, respectively, are also associated with genes involved in fatty acid metabolism. These results suggest a close link between milk fatty acid and protein metabolism. In goats, variants that are responsible for a decrease in *CSN1S1* gene expression were also associated with a decrease in fat content, probably due to disruption of the structure and secretion of fat globules [58]. A similar relationship was pointed out in cattle by Knutsen et al. [49], who found a major effect of the *PAEP* gene region on the C4:0 content of bovine milk, and Pausch et al. [53], who identified strong pleiotropic effects of variants located in the *CSN1S1* gene on fat and protein content. In addition, a strong association between PAEP and omega-3 fatty acids was observed by Boichard et al. [48]. All of these genes, which contain the top-ranked variants for, in particular, cheese yields and fatty acid composition, thus represent good candidates. Alone, they explained the largest part of the phenotypic variance captured in the present study for CY_DM_ and FC, i.e. around 16% out of 20%.

In addition to the PPARA TF, we highlight here other genes for putative regulators as well, such as *ASXL3* (*additional sex combs like 3, transcriptional regulator*) and *bta*-*mir*-*200c*, which interact with many genes of the network (276 and 240, respectively). Both are good candidates for key regulators in the network, as the protein encoded by *ASXL3* has been shown to negatively regulate lipogenesis and *bta*-*mir*-*200c* miRNA has been found to be highly expressed in the mammary gland [59–61] and present in milk whey [62]. Interestingly, all of the regulators that we identified in our study were different from the TF found in previous studies that applied similar approaches to study milk proteins [10] or fatty acids [9]. Unlike these studies, we analyzed here milk protein, fatty acid, and mineral composition as well as cheese-making traits all together, which might explain the identification of different regulatory pathways. However, in spite of this, some of the significantly enriched GO terms or KEGG pathways that we highlight here were concordant with those previously reported for CMP traits (Ca signaling pathway) [7], milk protein content (potassium ion transport) [10], or fatty acid content (hormone and steroid metabolic processes) [9].

### Causative variants

The approach that we used, which combines GWAS and post-GWAS analyses, was successful both in confirming previously reported candidate genes and in identifying new candidates that appear to be functionally linked to the analyzed traits. This was possible because our analyses were based on a large sample size, sequence-level genotypes, and detailed phenotypes for milk components in addition to complex CMP traits. However, for most of these genes, the top-ranked variant identified here was different both from what we had found before in an analysis of milk protein and fatty acid composition and from what had been detected in previous studies. Since the first GWAS on WGS imputed from the 1000 Bull Genomes reference population, in 2014 [4], to date published GWAS based on this approach have generally converged towards the same candidate genes but rarely towards the same best candidate variants in these genes. Using data from humans, Faye et al. [63] showed that when the causal variant is less accurately genotyped or imputed than one of its highly correlated neighboring variants, the neighboring variant can capture the association better than the causal variant. However, in our study, the HD SNP, imputed more accurately than sequence variants, were rarely the top variants of the peaks, with the noticeable exception in the *SCD* gene. For *SLC37A1*, the peak variant was more significant than variants already proposed in other studies and slightly better imputed. Nevertheless, we can anticipate that by accumulating bovine sequence data from different breeds and different populations, future runs of the 1000 Bull Genome Project will lead to better identification of causative variants by GWAS. More specifically, the expansion of the bovine sequence database should increase the accuracy of imputed genotypes and thus the probability of identifying the right variant. In addition, if GWAS analyses can be carried out in different breeds, meta-analyses should lead to a better resolution due to the linkage disequilibrium at shorter distances between breeds than within breed, and thus to a better discrimination of causal variants.

## Conclusions

By combining GWAS and AWM approaches at the whole-genome sequence level on milk cheese-making and composition traits predicted from MIR spectra, this study highlights candidate genes with major effects that are functionally related to milk composition. For most of these, we are able to propose some candidate variants that are likely to be either causative or in linkage disequilibrium with causative variants. In addition to providing a better understanding of the metabolic pathways involved in the genetic determinism of cheese-making traits, this study should make it possible to select a set of variants that explain a large part of the genetic variability of cheese-making traits. The increase in the number of cows for which both genotypes and phenotypes are available allows better detection of variants which could be included in genomic prediction to more accurately select animals with high genetic merit for CMP and finally improve the efficiency of the cheese-making process, which is of vital economic importance in the dairy industry.

## Additional files

**Additional file 1: Figure S1.** −log_10_(P) plotted against the position of variants on *Bos Taurus* autosomes for milk composition. Manhattan Plot obtained from GWAS for milk composition traits.

**Additional file 2: Table S1.** Description of the 264 genes located in confidence intervals of the QTL detected by GWAS for milk cheese-making and composition traits. Name and position of candidate genes identified by GWAS.

**Additional file 3: Table S2.** Percentage of the phenotypic variance of milk CMP and composition traits explained by each QTL. Individual effects of QTL on all CMP and composition traits, expressed as a percentage of the phenotypic variance of the trait.

**Additional file 4: Table S3.** Description of the 736 genes selected by the gene network analysis for milk cheese-making and composition traits. Name and position of candidate genes identified by AWM.

**Additional file 5: Table S4.** Gene ontology (GO) terms and KEGG pathways for genes selected by AWM. Name, description and list of genes of the gene ontology terms and KEGG pathways identified in the gene network analysis.

## Data Availability

The data (genotypes and phenotypes) that enabled the findings of this study were made available by UMOTEST, CEL25-90, and HSCEL. However, restrictions apply to the availability of these data: they were used under license for the current study, and are not publicly available.
