# Supplementary material for: Sequence-based GWAS, network and pathway analyses reveal genes co-associated with milk cheese-making properties and milk composition in Montbéliarde cows
Source: Genet Sel Evol. 2019 Jul 1;51:34. doi: 10.1186/s12711-019-0473-7 (PMC6604208; doi:10.1186/s12711-019-0473-7)
Supplement: Supplementary file 1 — Additional file 1: Figure S1. −log10(P) plotted against the position of variants on Bos Taurus autosomes for milk composition. Manhattan Plot obtained from GWAS for milk composition traits. [file 12711_2019_473_MOESM1_ESM.docx]

**Figure S1.** –log_10_(*P*) plotted against the position of variants on *Bos Taurus* autosomes for milk composition

1. Protein composition


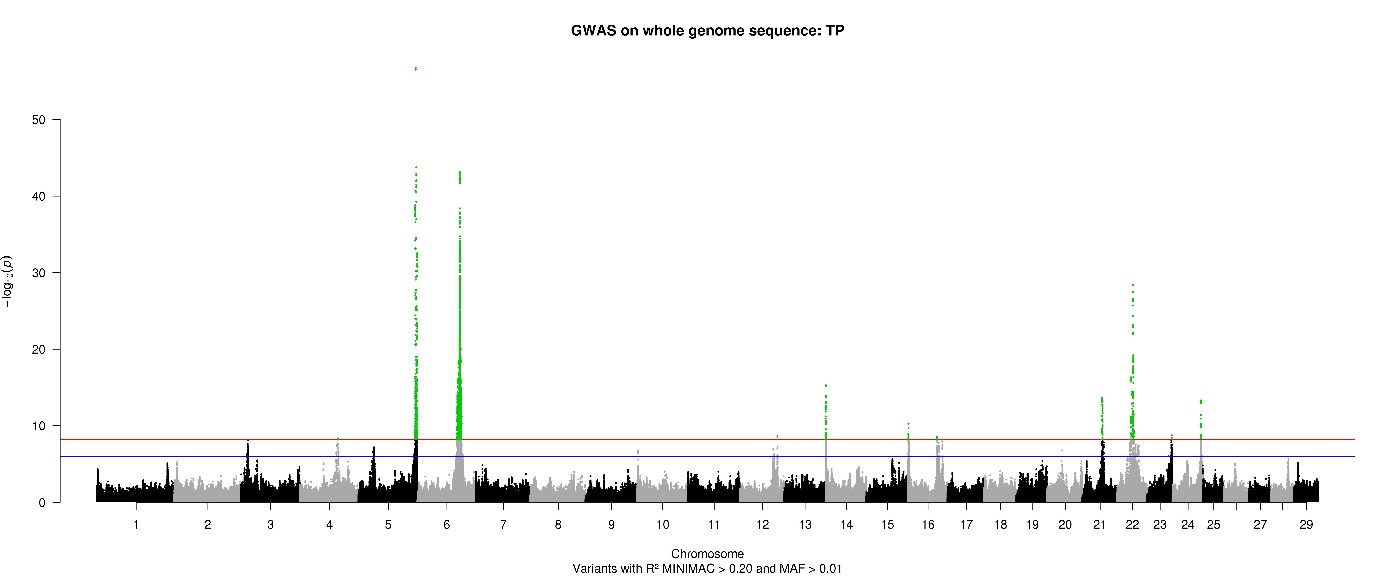


PC


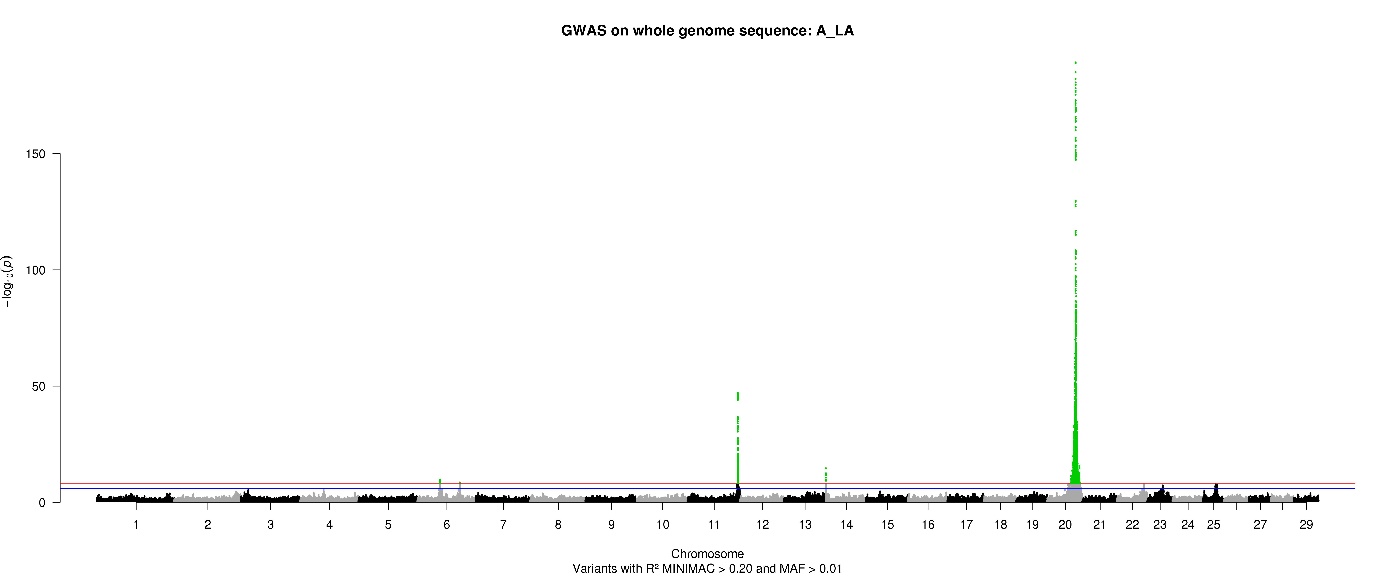


α-LA


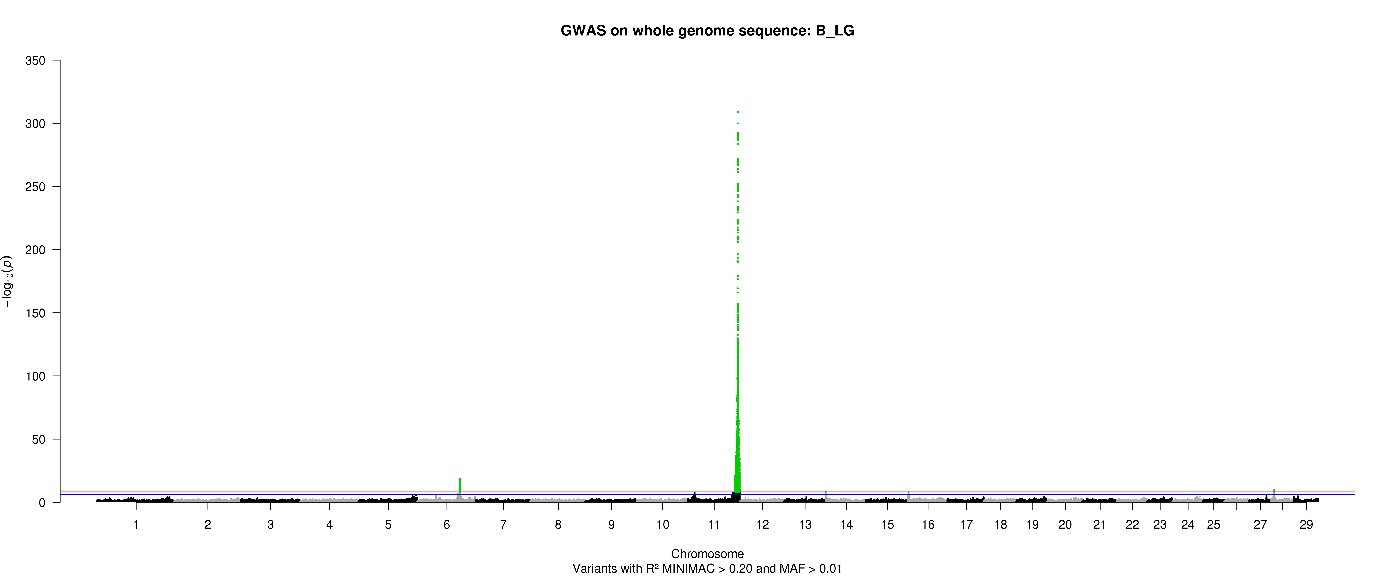


β-LG


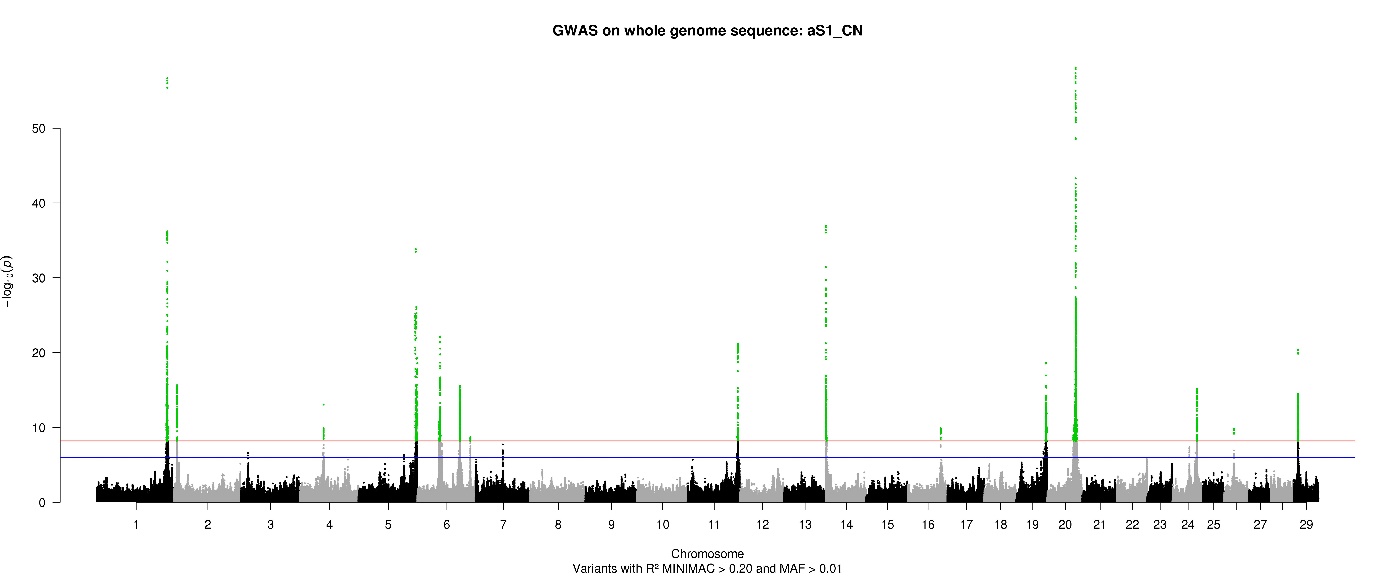

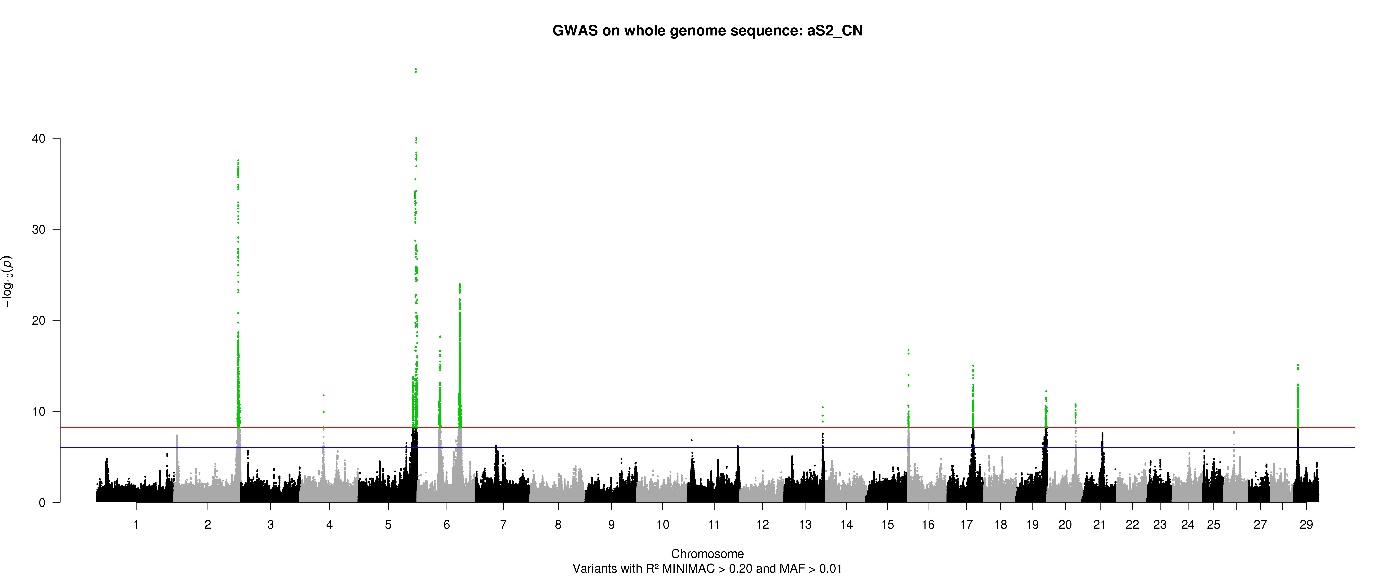

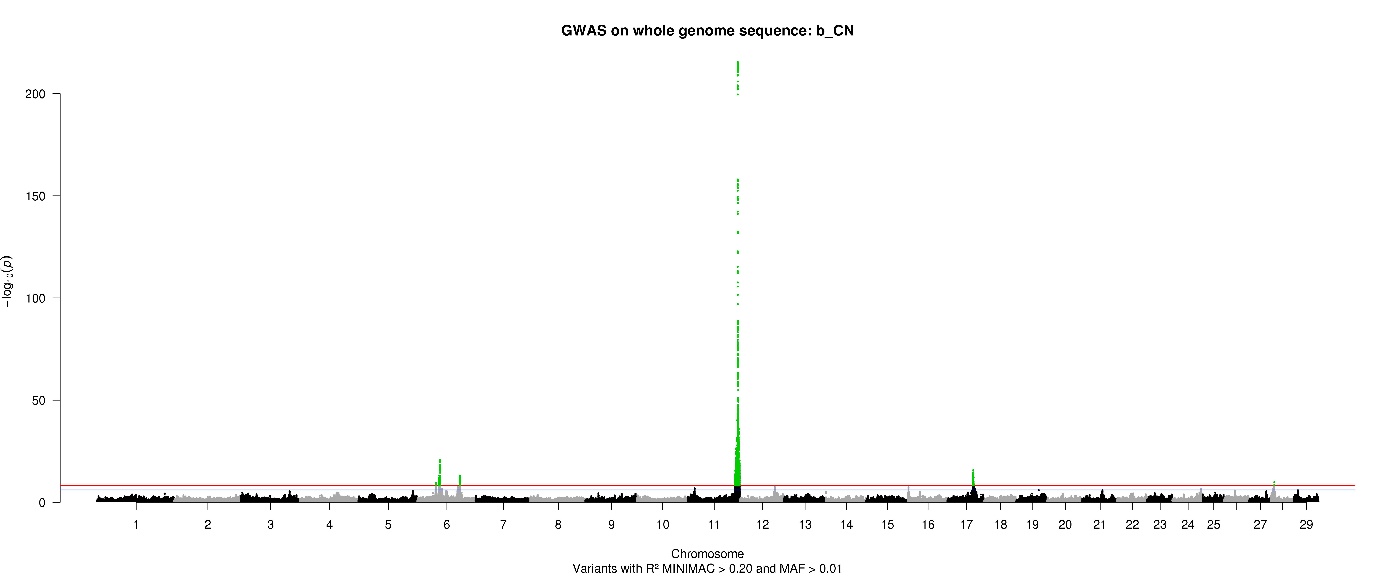

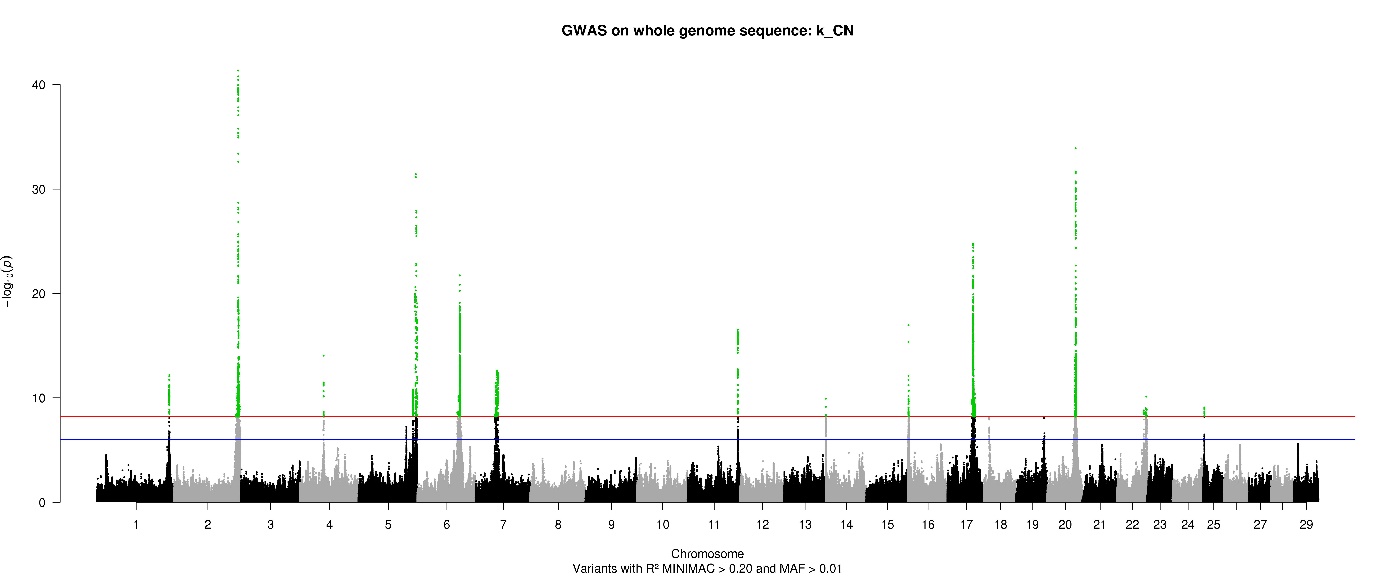


κ-CN

β-CN

αS2-CN

αS1-CN


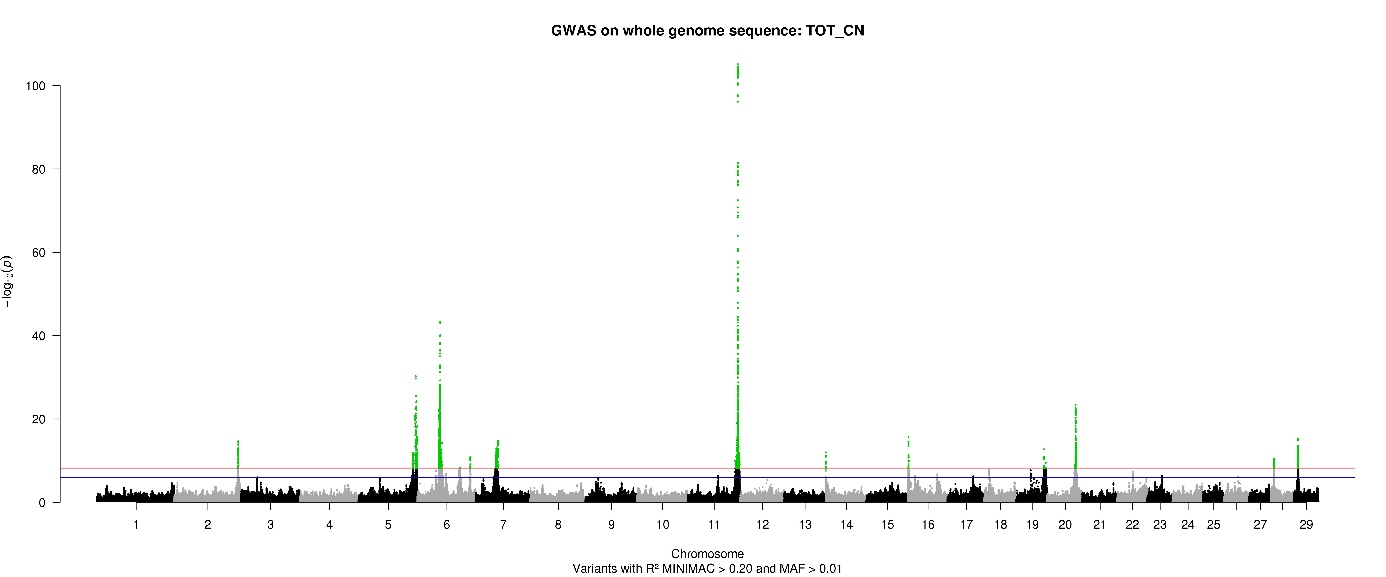

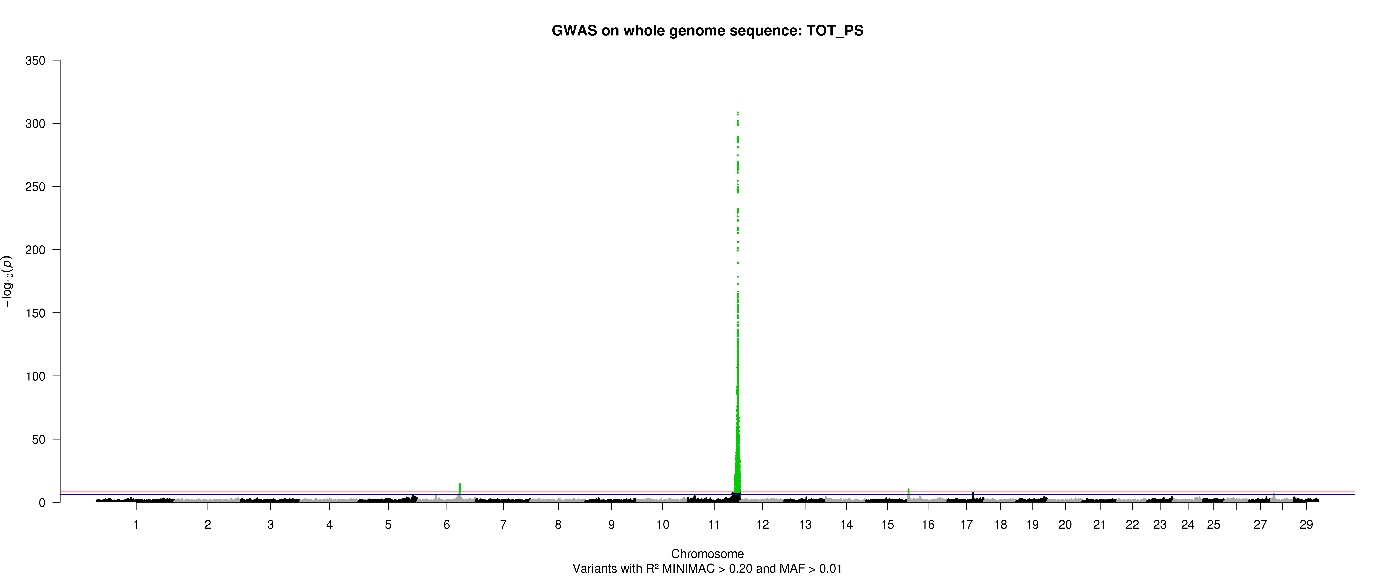


ΣCN

ΣWP

1. Fatty acid composition


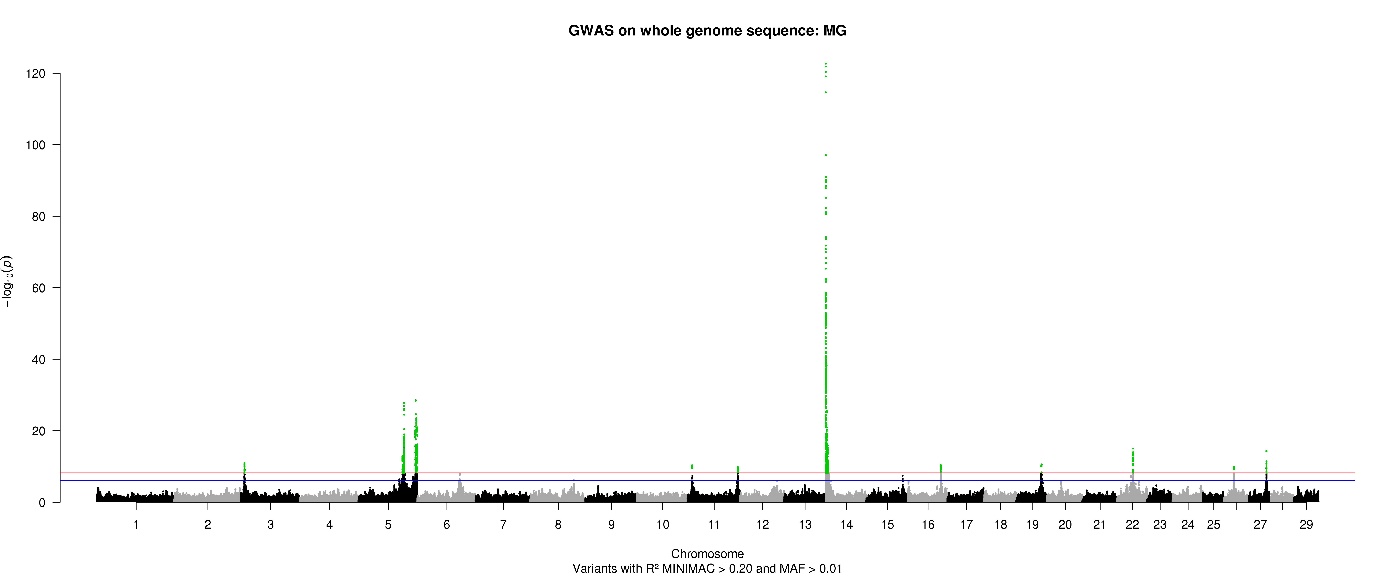


FC


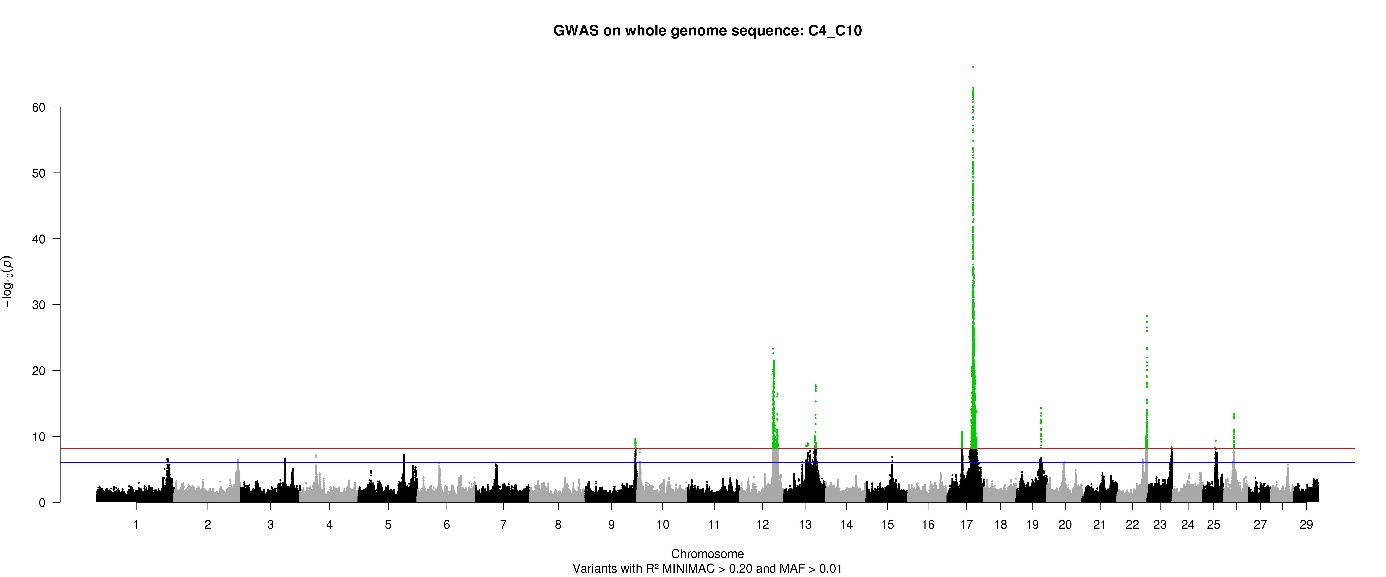

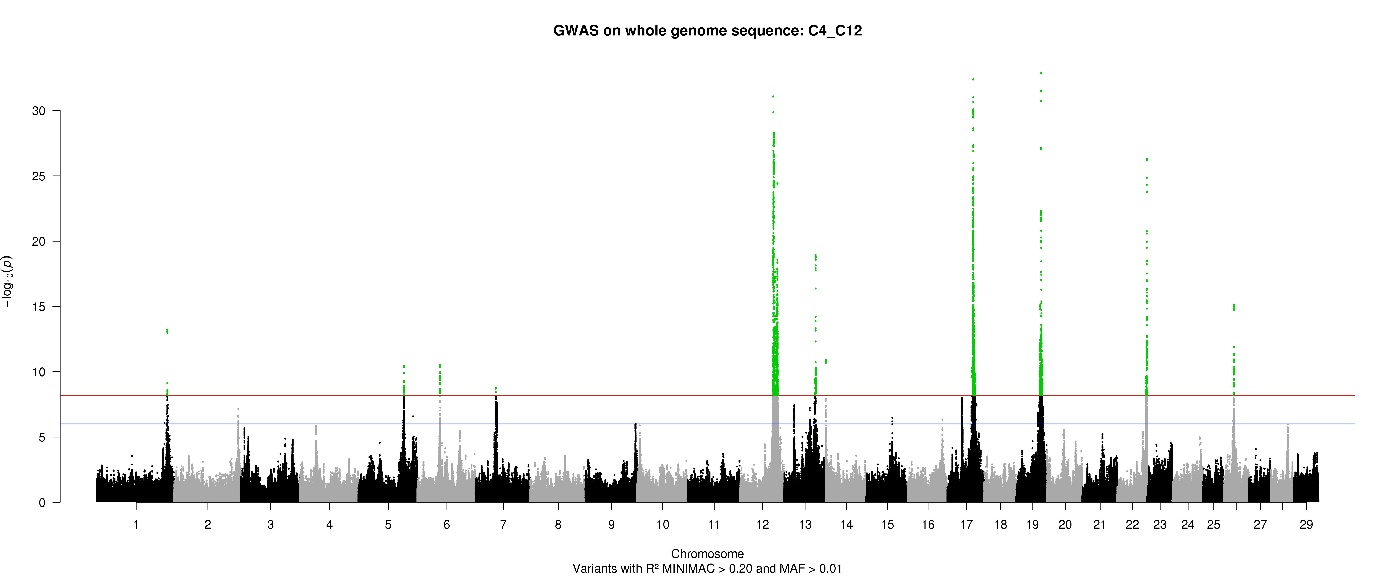

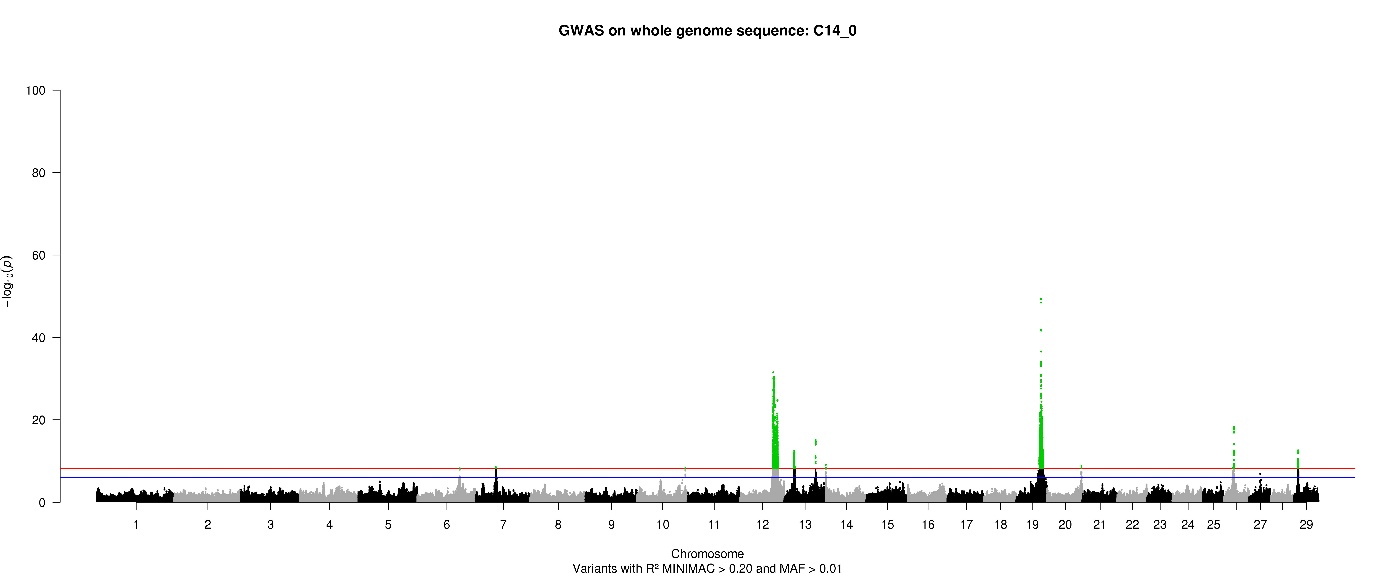

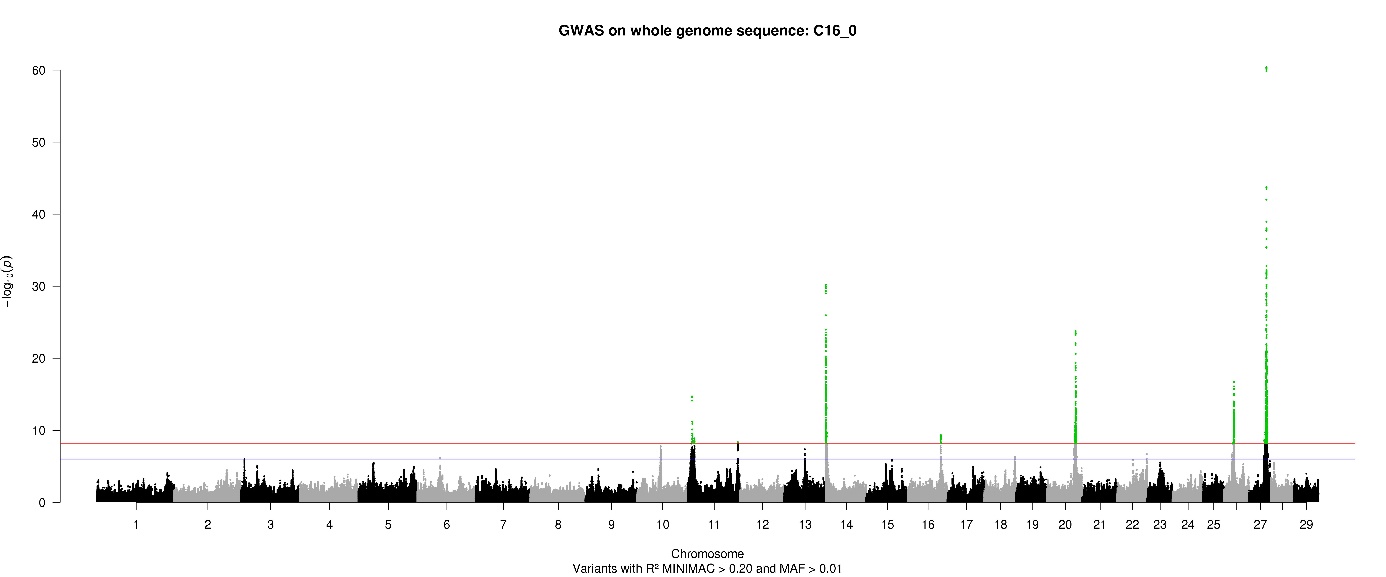


C16:0

C14:0

Σ C4-C12

Σ C4-C10


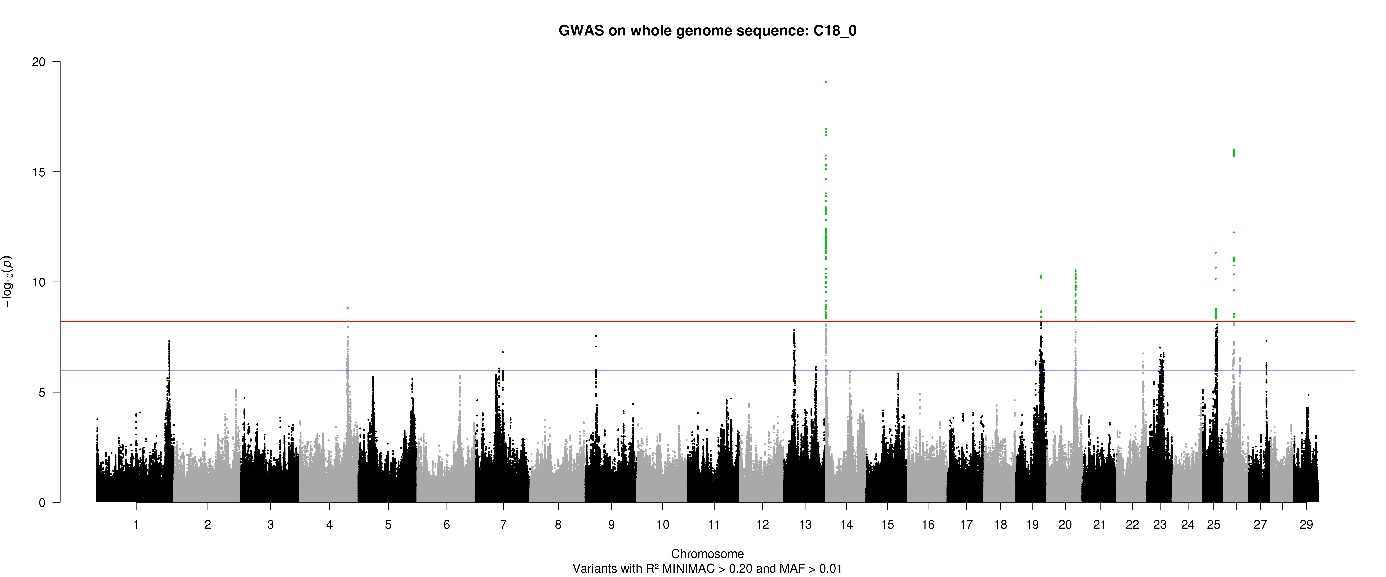

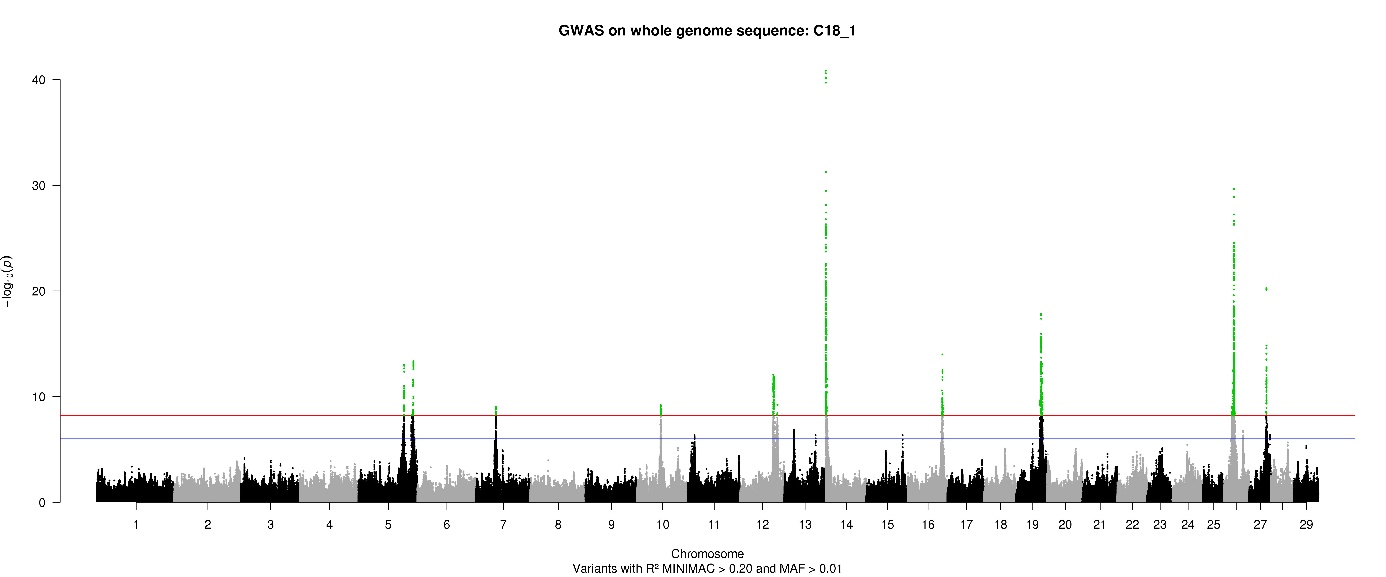

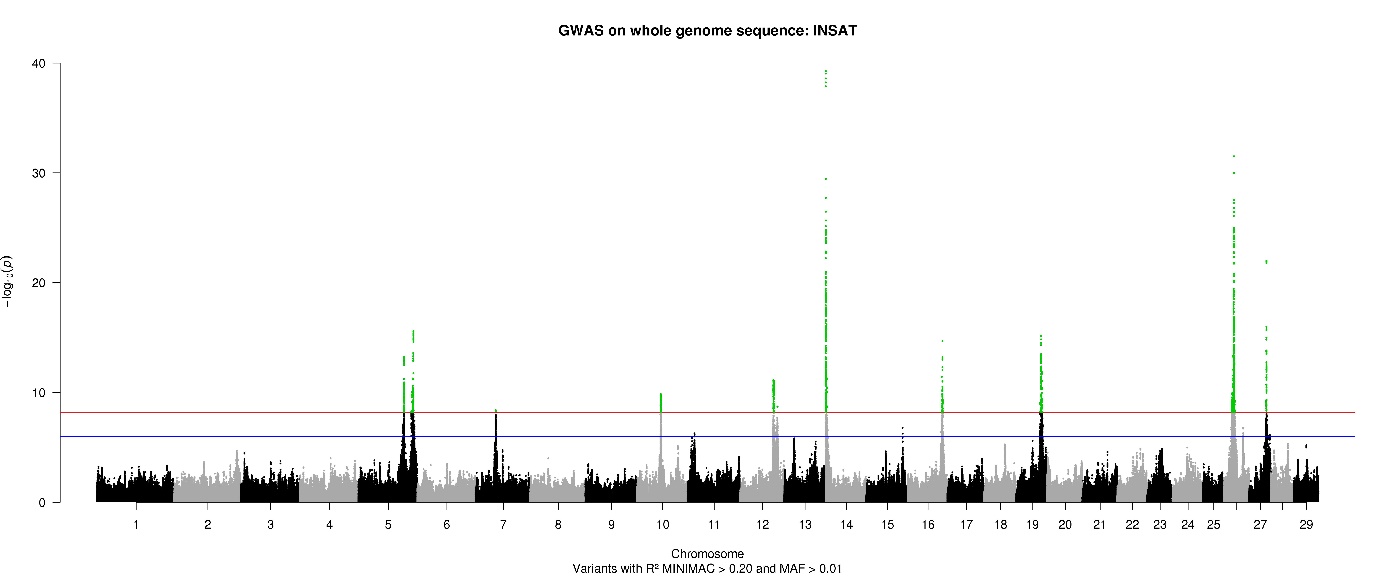

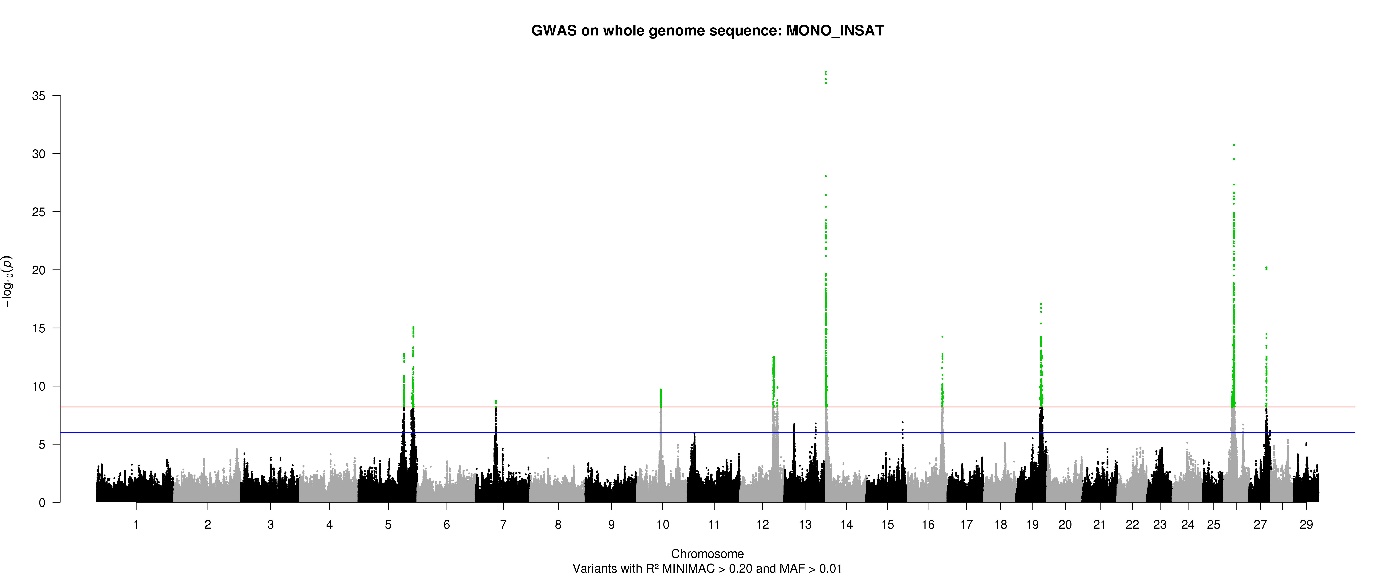


MUFA

UFA

C18:1

C18:0


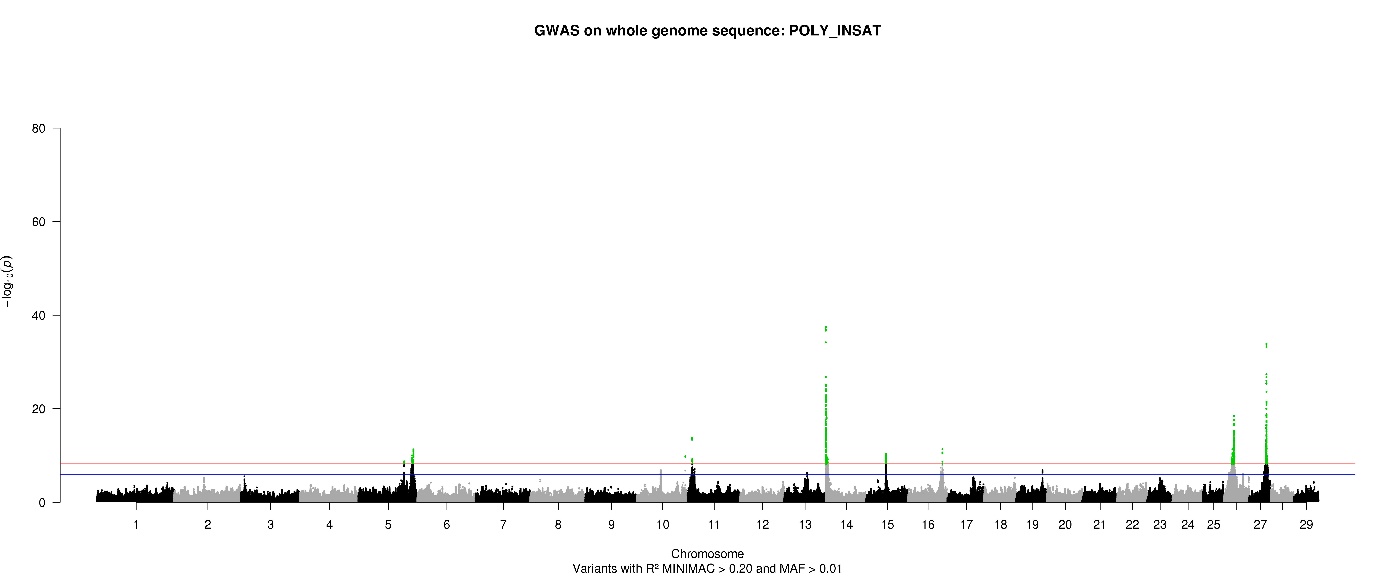

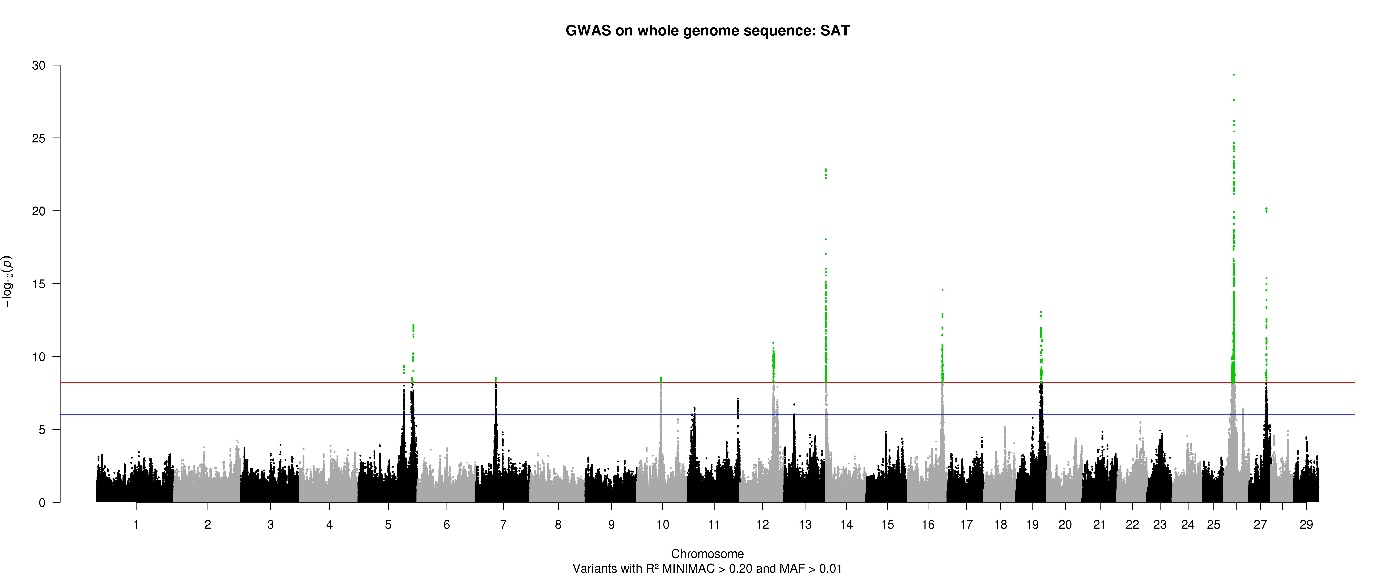


SFA

PUFA

1. Mineral composition


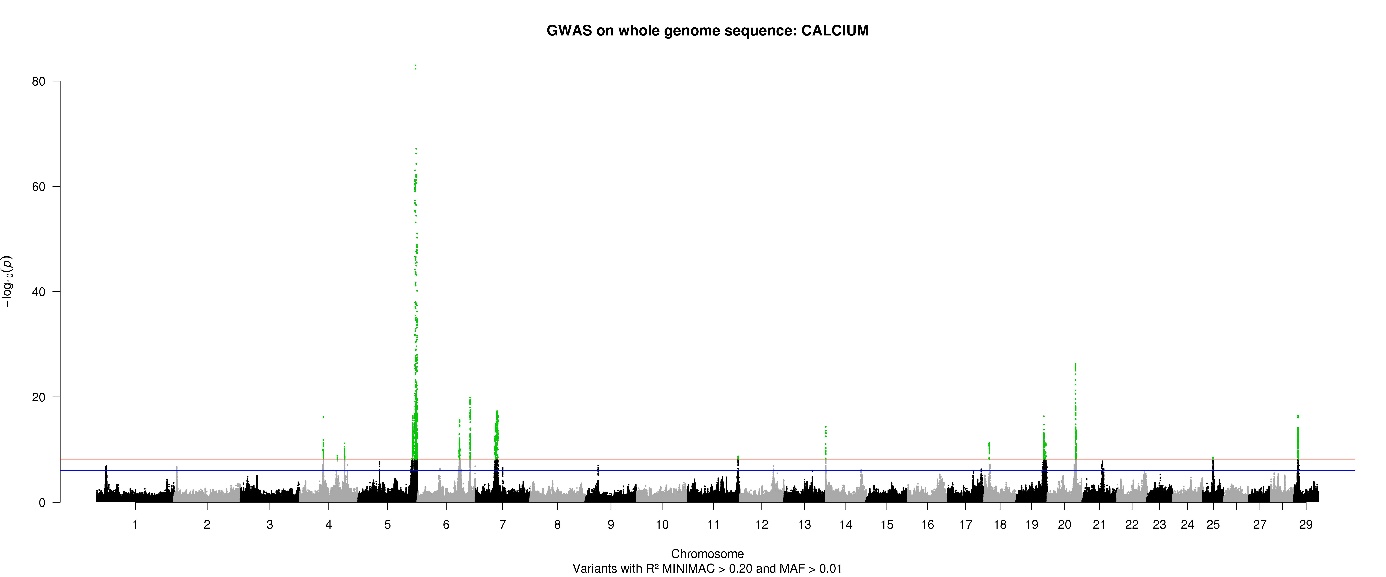

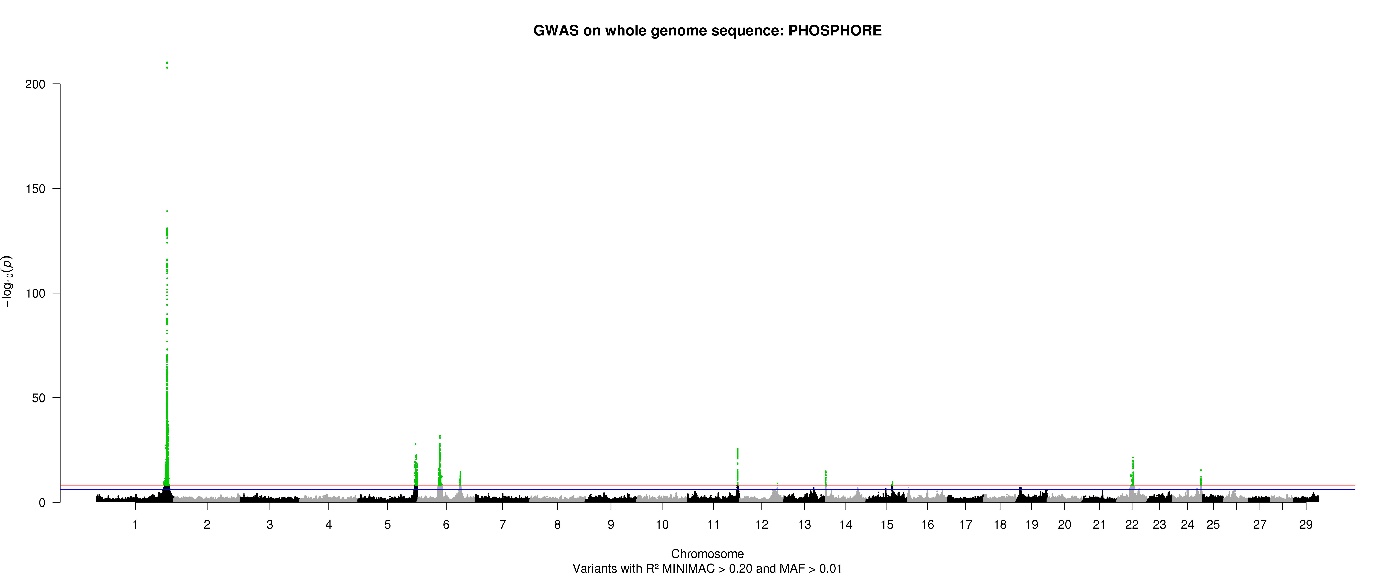

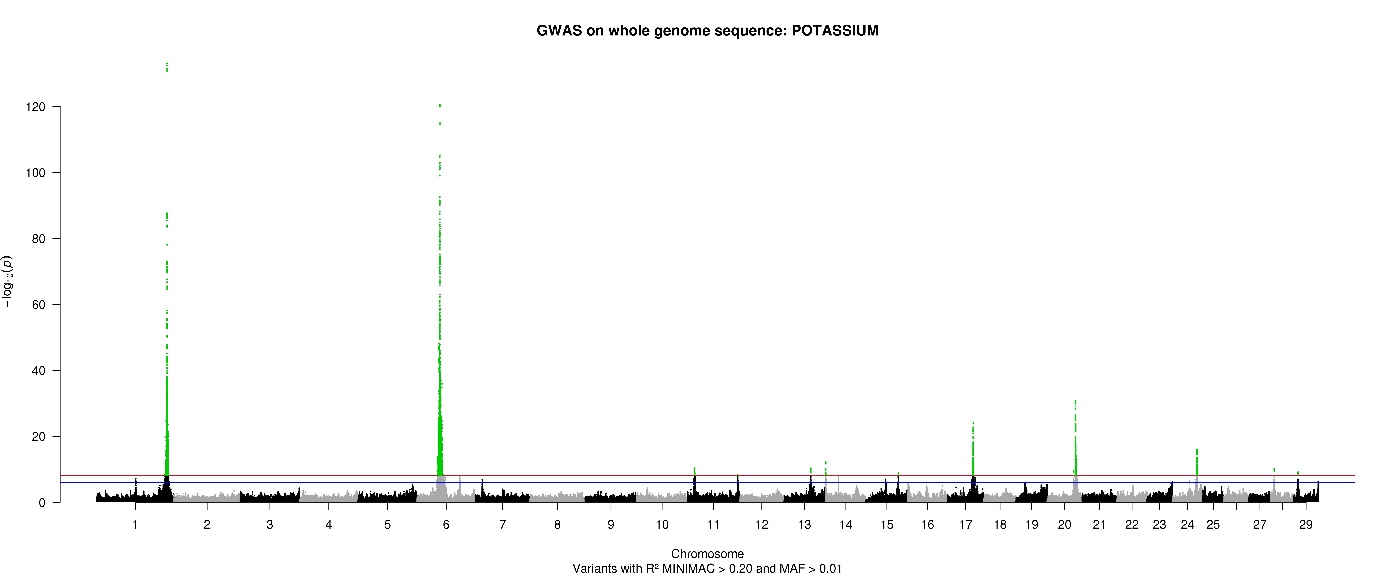


Potassium

Phosphore

Calcium


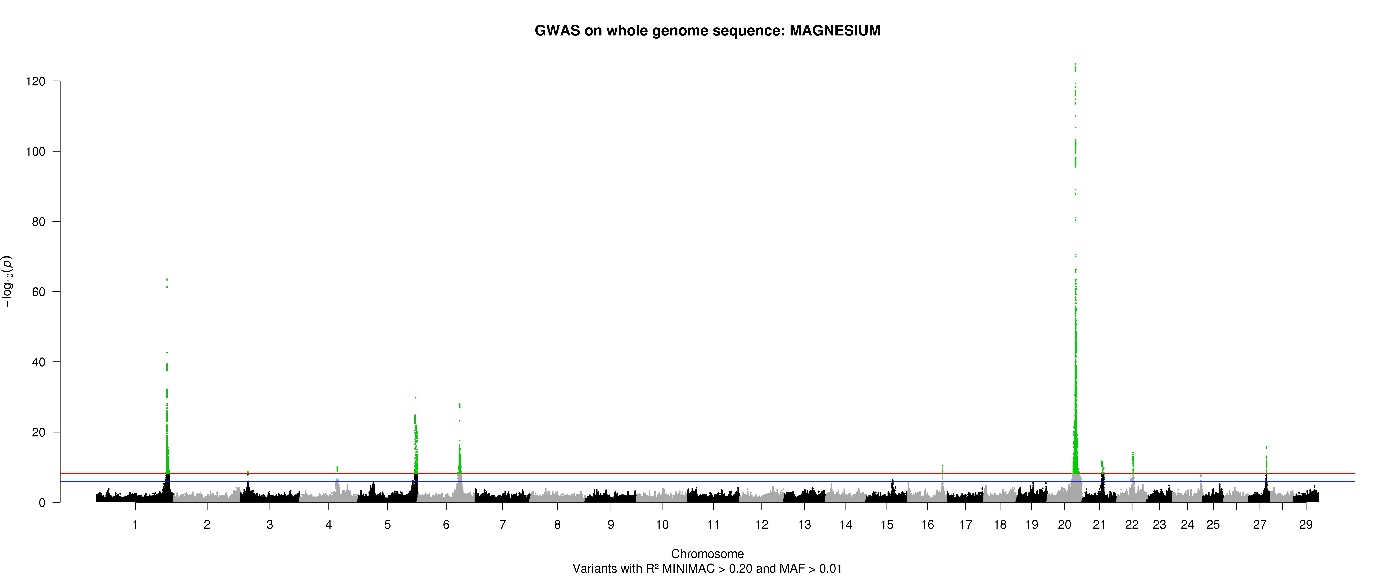

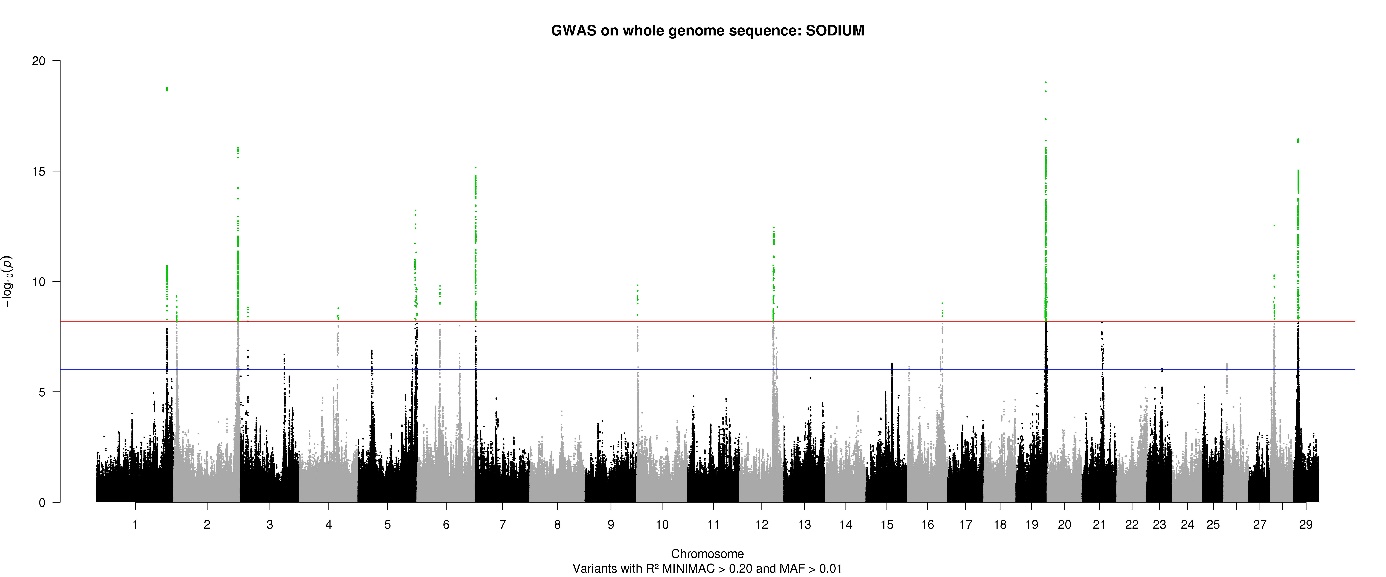


Sodium

Magnesium


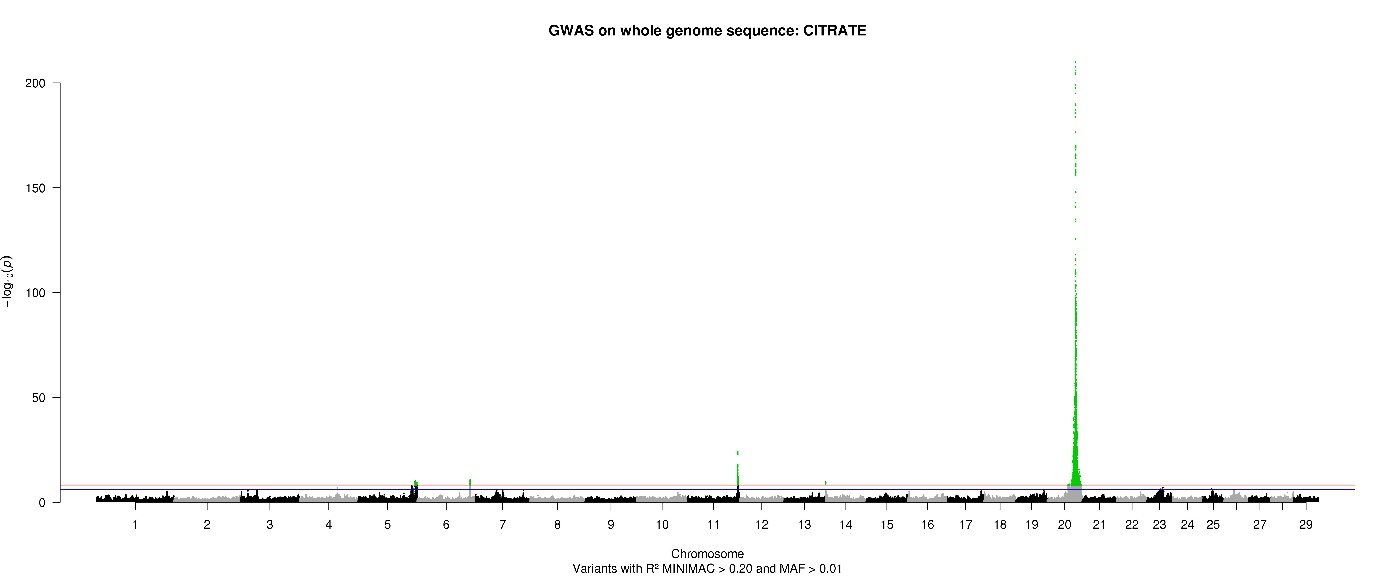

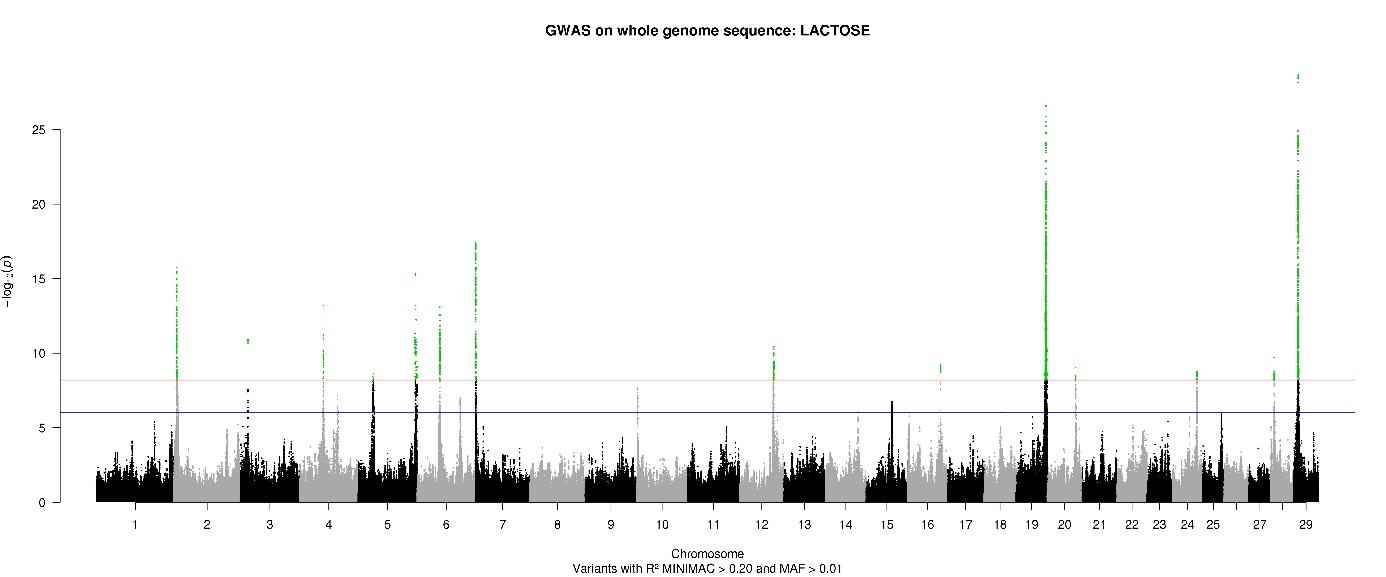


Lactose

Citrate
