# Supplementary material for: Sequence-based GWAS, network and pathway analyses reveal genes co-associated with milk cheese-making properties and milk composition in Montbéliarde cows
Source: Genet Sel Evol. 2019 Jul 1;51:34. doi: 10.1186/s12711-019-0473-7 (PMC6604208; doi:10.1186/s12711-019-0473-7)
Supplement: Supplementary file 3 — Additional file 3: Table S2. Percentage of the phenotypic variance of milk CMP and composition traits explained by each QTL. Individual effects of QTL on all CMP and composition traits, expressed as a percentage of the phenotypic variance of the trait. [file 12711_2019_473_MOESM3_ESM.docx]

**Table S2. Percentage of the phenotypic variance of milk CMP and composition traits explained by each QTL**

|  | **CMP** | | | | | | | | | **Proteins** | | | | | | | | | **Fatty acids** | | | | | | | | | | | **Minerals** | | | | |  |  |  |  |
| --- | --- | --- | --- | --- | --- | --- | --- | --- | --- | --- | --- | --- | --- | --- | --- | --- | --- | --- | --- | --- | --- | --- | --- | --- | --- | --- | --- | --- | --- | --- | --- | --- | --- | --- | --- | --- | --- | --- |
| **QTL** | **CY_FRESH_** | **CY_DM_** | **CY_FAT-PROT_** | **a_PCC_** | **K10/RCT_PCC_** | **a_SC_** | **a2_SC_** | **K10/RCT_SC_** | **pH_0_PCC_** | **PC** | **α-LA** | **β-LG** | **αs1-CN** | **αs2-CN** | **β-CN** | **κ-CN** | **ΣCN** | **ΣWP** | **FC** | **SFA** | **MUFA** | **UFA** | **PUFA** | **Σ C4-C10** | **Σ C4-C12** | **C14:0** | **C16:0** | **C18:0** | **C18:1** | **Ca** | **P** | **Mg** | **K** | **Na** | **Lactose** | **Citrate** | **Mean / QTL** | **# Traits** |
| 1 |  |  | 0.3 | 0.5 |  |  |  | 0.2 | 2.8 |  |  |  | 2.8 |  |  |  |  |  |  |  |  |  |  | 0.3 | 0.6 |  |  |  |  |  | 10.5 | 3.2 | 4.9 | 0.8 |  |  | 2.44 | 11 |
| 2 | 0.2 |  |  |  |  | 0.4 | 0.3 |  | 0.4 |  |  |  | 0.6 | 1.1 |  |  |  |  |  |  |  |  |  |  |  |  |  |  |  | 0.3 |  |  |  | 0.6 | 1.9 |  | 0.65 | 9 |
| 3 |  |  |  | 0.2 |  |  |  |  |  |  |  |  |  | 1.6 |  | 1.9 | 0.6 |  |  |  |  |  |  |  |  |  |  |  |  |  |  |  |  | 0.5 |  |  | 0.96 | 5 |
| 4 | 0.8 | 0.9 |  |  |  |  |  |  |  |  |  |  |  |  |  |  |  |  | 1.0 |  |  |  |  |  |  |  |  |  |  |  |  |  |  |  |  |  | 0.90 | 3 |
| 5 |  |  |  |  |  | 0.3 |  |  |  | 0.6 |  |  |  |  |  |  |  |  |  |  |  |  |  |  |  |  |  |  |  |  |  | 1.3 |  | 0.2 | 0.2 |  | 0.50 | 5 |
| 6 |  |  |  | 0.8 |  | 1.0 |  |  |  | 0.7 |  |  |  |  |  |  |  |  |  |  |  |  |  |  |  |  |  |  |  |  |  |  |  |  |  | 1.4 | 0.97 | 4 |
| 7 |  |  |  |  |  |  | 0.9 |  | 4.8 |  |  |  | 1.5 | 1.3 |  | 1.7 |  |  |  |  |  |  |  |  |  |  |  |  |  | 1.9 |  |  |  |  | 1.5 |  | 1.93 | 7 |
| 8 |  |  |  |  |  |  |  |  |  | 0.3 |  |  |  |  |  |  |  |  |  |  |  |  |  |  |  |  |  |  |  | 0.4 |  | 0.5 |  | 0.5 | 1.2 | 0.3 | 0.54 | 6 |
| 9 |  |  |  |  |  |  |  |  |  |  |  |  |  |  |  |  |  |  |  |  |  |  |  |  |  |  |  | 0.4 |  | 0.9 |  |  |  |  |  |  | 0.66 | 2 |
| 10 |  |  |  |  | 0.4 |  |  | 0.4 |  | 0.6 |  |  |  |  |  |  |  |  |  |  |  |  |  |  |  |  |  |  |  |  |  | 0.3 |  | 0.2 | 0.4 |  | 0.38 | 6 |
| 11 | 0.5 | 0.7 | 0.4 |  |  |  |  |  |  |  |  |  | 0.2 |  |  |  |  |  | 1.2 | 0.2 | 0.3 | 0.3 | 0.1 | 0.2 | 0.4 |  |  |  | 0.3 |  |  |  |  |  |  |  | 0.41 | 12 |
| 12 | 1.1 | 1.1 | 0.5 |  | 1.8 | 0.2 | 1.7 | 1.2 |  | 2.0 |  |  | 1.1 | 1.5 |  | 1.1 | 0.9 |  | 1.1 |  |  |  |  |  |  |  |  |  |  | 2.7 | 1.2 | 1.4 |  | 0.4 | 0.5 | 0.5 | 1.15 | 19 |
| 13 |  |  | 0.2 |  |  |  |  |  |  |  |  |  |  |  | 1.2 |  | 1.0 |  |  |  |  |  |  |  |  |  |  |  |  |  |  |  |  |  |  |  | 0.80 | 3 |
| 14 | 0.9 |  | 1.1 | 1.8 |  | 1.0 |  | 0.9 | 4.6 |  | 0.5 |  | 1.1 | 0.9 | 4.0 |  | 2.0 |  |  |  |  |  |  |  | 0.8 |  |  |  |  | 0.4 | 1.6 |  | 4.6 | 0.4 | 2.7 |  | 1.72 | 17 |
| 15 | 2.9 | 2.8 | 3.2 | 1.0 | 20.1 | 1.9 | 17.2 | 19.8 |  | 5.6 | 0.7 | 1.2 | 1.1 | 2.0 | 0.9 | 2.3 | 0.7 | 0.8 |  |  |  |  |  |  |  | 0.5 |  |  |  | 0.7 |  | 1.5 |  |  |  |  | 4.34 | 20 |
| 16 |  |  |  | 0.2 |  | 0.5 |  |  | 0.7 |  |  |  | 0.3 |  |  |  | 0.4 |  |  |  |  |  |  |  |  |  |  |  |  | 0.7 |  |  |  |  |  | 0.5 | 0.47 | 7 |
| 17 |  |  |  |  |  |  |  |  |  |  |  |  |  |  |  |  |  |  |  |  |  |  |  |  |  |  |  |  |  |  |  |  |  | 0.9 | 0.6 |  | 0.76 | 2 |
| 18 |  |  | 0.5 | 1.3 |  | 1.3 |  | 0.5 |  |  |  |  |  | 0.5 |  | 1.0 | 1.0 |  |  |  |  |  |  |  |  |  |  |  |  | 1.6 |  |  |  |  |  |  | 0.96 | 8 |
| 19 |  |  |  |  |  |  |  |  |  |  |  |  |  |  |  |  |  |  |  |  |  |  |  | 0.2 |  |  |  |  |  |  |  |  |  |  |  |  | 0.25 | 1 |
| 20 |  |  |  |  |  | 0.2 |  |  |  | 0.3 |  |  |  |  |  |  |  |  |  |  |  |  |  |  |  |  |  |  |  |  |  |  |  | 0.4 | 0.3 |  | 0.28 | 4 |
| 21 |  |  |  |  |  |  |  |  |  |  |  |  |  |  |  |  |  |  |  | 0.2 | 0.4 | 0.2 | 0.2 |  |  |  | 0.6 |  | 0.4 |  |  |  |  |  |  |  | 0.34 | 6 |
| 22 |  |  |  |  |  |  |  |  |  |  |  |  |  |  |  |  |  |  |  |  |  |  | 0.5 |  |  | 0.3 |  |  |  |  |  |  |  |  |  |  | 0.40 | 2 |
| 23 | 0.5 | 0.6 | 0.6 |  |  |  |  |  |  |  |  |  |  |  |  |  |  |  | 0.6 | 0.2 |  | 0.2 | 0.3 |  |  |  | 0.5 |  | 0.2 |  |  |  |  |  |  |  | 0.41 | 9 |
| 24 |  |  |  |  |  |  |  |  | 3.6 |  |  | 0.5 |  |  | 0.2 |  |  | 0.4 |  | 0.1 |  | 0.1 |  |  |  |  |  |  | 0.1 |  |  |  | 1.0 |  |  |  | 0.76 | 8 |
| 25 |  |  |  |  |  |  |  |  | 2.8 |  |  |  |  |  |  |  |  |  |  |  |  |  |  |  |  |  |  |  |  |  |  |  |  |  |  |  | 2.83 | 1 |
| 26 | 3.2 | 4.0 | 8.5 | 4.4 | 1.0 | 1.3 | 1.4 | 1.5 |  |  | 2.8 | 59.2 | 1.1 | 0.5 | 12.1 | 1.3 | 7.5 | 56.5 | 0.7 | 0.2 |  |  |  |  |  |  | 0.3 |  |  |  | 2.3 |  | 0.3 |  |  | 2.0 | 7.82 | 22 |
| 27 | 0.3 |  | 0.2 |  |  |  |  |  |  | 0.4 |  |  |  |  |  |  |  |  |  | 0.3 | 1.0 | 0.4 |  | 1.2 | 1.6 | 1.0 |  |  | 0.4 | 0.4 | 0.4 |  |  | 0.8 | 0.7 |  | 0.65 | 14 |
| 28 |  |  |  |  |  |  |  |  |  |  |  |  |  |  |  |  |  |  |  | 0.2 | 0.6 | 0.2 |  |  | 0.8 | 0.3 |  | 0.5 | 0.6 |  |  |  |  |  |  |  | 0.45 | 7 |
| 29 |  |  |  |  |  |  |  |  | 0.7 |  |  |  |  |  |  |  |  |  |  |  |  |  |  | 0.5 |  |  |  |  |  |  |  |  |  |  |  |  | 0.62 | 2 |
| 30 |  |  |  |  |  |  |  |  |  |  |  |  |  |  |  |  |  |  |  |  |  |  |  | 0.4 | 0.4 |  |  |  |  |  |  |  | 0.4 |  |  |  | 0.43 | 3 |
| 31 |  |  |  |  |  |  |  |  |  |  |  |  |  |  |  |  |  |  |  |  | 0.3 | 0.2 |  | 1.2 | 1.3 | 0.6 |  |  | 0.3 |  |  |  |  |  |  |  | 0.66 | 6 |
| 32 |  |  |  |  |  |  |  |  |  |  |  |  |  | 1.6 |  |  |  |  |  |  |  |  |  |  |  |  |  |  |  |  |  |  |  |  |  |  | 1.63 | 1 |
| 33 | 4.8 | 6.4 | 1.0 |  | 1.6 |  | 1.5 | 1.1 |  | 1.0 | 0.7 | 0.7 | 2.9 |  |  | 1.1 | 1.1 |  | 11.9 | 1.1 | 2.0 | 2.1 | 1.3 |  | 0.8 | 0.5 | 2.2 | 1.4 | 2.3 | 2.1 | 1.0 |  | 1.0 |  |  | 1.0 | 2.10 | 26 |
| 34 |  |  |  |  |  |  |  |  |  |  |  |  |  |  |  |  |  |  |  |  |  |  | 0.3 |  |  |  |  |  |  |  |  |  | 0.4 |  |  |  | 0.34 | 2 |
| 35 |  |  |  |  |  |  |  |  |  |  |  |  |  |  |  |  |  |  |  |  |  |  |  | 0.4 | 0.4 |  |  |  |  |  | 0.7 |  |  |  | 0.6 |  | 0.52 | 4 |
| 36 |  |  |  | 0.2 |  | 0.9 |  |  |  | 0.4 |  | 0.2 |  | 0.9 | 0.2 | 1.1 | 0.4 | 0.2 |  |  |  |  |  |  |  |  |  |  |  |  | 0.2 | 0.2 |  | 0.1 |  |  | 0.43 | 12 |
| 37 | 0.2 | 0.2 |  | 0.1 | 0.3 | 0.3 | 0.2 | 0.3 |  | 0.3 |  |  |  |  |  |  | 0.2 |  |  |  |  |  |  |  |  |  |  |  |  |  |  |  |  |  |  |  | 0.23 | 9 |
| 38 | 0.3 | 0.4 |  |  |  |  |  |  |  |  |  |  | 0.5 |  |  |  |  |  | 0.4 |  |  |  |  |  | 0.3 |  | 0.2 |  |  |  |  |  |  |  | 0.5 |  | 0.37 | 7 |
| 39 |  |  |  |  | 1.1 |  | 0.9 | 1.0 |  | 1.4 |  |  |  |  |  |  |  |  |  | 1.0 | 1.1 | 1.1 | 0.5 |  | 0.4 |  |  |  | 1.1 |  | 0.6 | 1.8 | 0.5 | 1.2 |  |  | 0.99 | 14 |
| 40 |  |  |  |  |  |  |  |  |  |  |  |  |  |  |  |  |  |  |  |  |  |  |  | 1.5 | 1.6 |  |  |  |  |  |  |  |  |  |  |  | 1.53 | 2 |
| 41 |  |  |  |  | 0.9 |  | 0.6 | 0.5 |  |  |  |  |  | 0.6 | 1.5 | 1.0 | 0.3 | 0.2 |  |  |  |  |  | 3.3 |  |  |  |  |  |  |  |  | 1.0 |  |  |  | 0.99 | 10 |
| 42 |  |  |  | 0.5 |  | 0.2 |  |  |  |  |  |  |  |  |  | 0.8 | 0.2 |  |  |  |  |  |  |  |  |  |  |  |  | 0.5 |  |  |  |  |  |  | 0.44 | 5 |
| 43 | 0.5 | 0.7 |  |  |  |  |  |  |  |  |  |  |  |  |  |  |  |  | 1.2 | 0.5 | 0.5 | 0.4 |  | 0.6 | 1.4 | 1.5 |  | 0.7 | 0.5 |  |  |  |  |  |  |  | 0.79 | 11 |
| 44 |  |  |  | 0.4 |  | 0.3 |  |  |  |  |  |  |  | 0.3 |  | 0.7 | 0.6 |  |  | 0.1 | 0.1 | 0.1 |  |  | 0.3 | 0.3 |  | 0.1 | 0.2 | 0.8 |  |  |  |  |  |  | 0.33 | 13 |
| 45 |  |  |  | 0.2 |  |  |  |  |  |  |  |  | 0.4 | 1.5 |  |  | 0.2 |  |  |  |  |  |  |  |  | 0.1 |  |  |  | 0.2 |  |  |  | 0.4 | 0.6 |  | 0.47 | 8 |
| 46 | 0.2 |  | 0.5 | 0.2 |  | 1.1 |  | 0.4 | 0.4 |  | 17.6 |  | 8.7 | 1.5 |  | 7.4 | 1.1 |  |  |  |  |  |  |  |  |  | 2.6 | 1.1 |  | 1.1 |  | 20.5 | 3.9 |  | 0.4 | 31.6 | 5.56 | 18 |
| 47 |  |  |  |  | 1.5 |  | 0.4 | 0.4 |  | 1.8 |  |  |  | 1.0 | 0.4 | 0.2 |  |  |  |  |  |  |  |  |  |  |  |  |  | 1.2 |  | 1.3 |  | 0.7 |  |  | 0.90 | 10 |
| 48 | 0.7 | 0.9 | 0.3 | 0.1 | 0.9 | 0.4 | 0.6 | 0.8 |  | 1.4 |  |  |  |  |  |  | 0.4 |  | 0.8 |  |  |  |  |  |  |  | 0.2 |  |  |  | 0.9 | 0.6 |  |  |  |  | 0.64 | 14 |
| 49 |  |  |  |  |  | 0.3 |  |  | 0.6 |  | 0.3 |  |  |  |  | 0.3 |  |  |  |  |  |  |  |  |  |  |  |  |  |  |  |  |  |  |  |  | 0.35 | 4 |
| 50 |  |  |  |  |  |  | 0.2 |  |  |  |  |  | 0.2 |  |  | 0.2 |  |  |  |  |  |  |  | 0.7 | 0.7 |  | 0.2 |  |  |  |  |  |  |  |  |  | 0.37 | 6 |
| 51 |  |  |  |  |  | 0.2 |  |  | 1.4 |  |  |  | 1.3 |  |  |  |  |  |  |  |  |  |  |  |  |  |  |  |  |  | 0.6 |  | 1.3 |  | 0.4 |  | 0.88 | 6 |
| 52 | 0.2 | 0.2 |  |  | 0.3 | 0.3 | 0.2 | 0.2 |  | 0.5 |  |  |  |  | 0.2 |  |  |  |  |  |  |  |  |  |  |  |  |  |  |  | 0.5 | 0.3 |  |  |  |  | 0.28 | 10 |
| 53 |  |  |  |  |  |  |  |  |  |  |  |  |  |  |  | 0.7 |  |  |  |  |  |  |  |  |  |  |  |  |  |  |  |  |  |  |  |  | 0.71 | 1 |
| 54 |  |  |  |  |  |  |  |  |  |  | 0.4 |  |  |  |  |  |  |  |  |  |  |  |  | 0.5 |  |  |  | 0.3 |  | 0.5 |  |  |  |  |  |  | 0.42 | 4 |
| 55 | 0.2 | 0.2 |  |  |  |  |  |  |  |  |  |  | 0.3 | 0.2 |  |  |  |  | 0.5 | 1.0 | 1.2 | 1.2 | 0.4 | 0.5 | 0.6 | 0.4 | 1.1 | 0.3 | 2.0 |  |  |  |  |  |  | 0.7 | 0.69 | 16 |
| 56 |  |  |  | 0.2 |  |  |  |  |  |  |  |  |  |  |  |  |  |  |  |  |  |  |  |  |  |  |  | 0.2 |  |  |  |  |  |  |  |  | 0.22 | 2 |
| 57 | 0.7 | 1.0 | 0.6 |  |  |  |  |  | 0.4 |  |  | 0.3 |  |  | 0.3 |  |  |  | 1.1 | 0.9 | 1.0 | 1.1 | 1.0 |  |  |  | 3.2 | 0.9 | 1.0 |  |  | 1.1 |  |  |  |  | 0.98 | 15 |
| 58 |  |  |  | 0.1 |  |  |  |  | 2.0 |  |  | 0.2 |  |  | 0.2 |  | 0.2 | 0.2 |  |  |  |  |  |  |  |  |  |  |  |  |  |  | 0.2 | 0.3 | 0.2 |  | 0.41 | 9 |
| 59 |  |  |  |  |  |  |  |  |  |  |  |  | 1.4 | 1.0 | 0.3 | 0.3 | 0.8 |  |  |  |  |  |  |  |  | 1.0 |  |  |  | 1.1 |  |  | 0.4 | 2.1 | 5.2 |  | 1.36 | 10 |
| Sum / Trait | 18.2 | 20.0 | 17.9 | 12.2 | 29.9 | 12.2 | 26.1 | 29.2 | 25.3 | 17.2 | 23.0 | 62.4 | 25.2 | 17.9 | 21.5 | 23.1 | 19.7 | 58.3 | 20.5 | 6.0 | 8.7 | 7.7 | 4.7 | 11.7 | 12.3 | 6.6 | 11.1 | 6.0 | 9.5 | 17.5 | 20.4 | 33.9 | 19.9 | 10.5 | 18.0 | 37.9 |  |  |
| # QTL | 18 | 14 | 14 | 17 | 11 | 19 | 13 | 15 | 13 | 15 | 7 | 7 | 17 | 17 | 12 | 17 | 19 | 6 | 11 | 13 | 11 | 13 | 9 | 14 | 16 | 11 | 10 | 10 | 13 | 18 | 12 | 13 | 13 | 17 | 17 | 8 |  |  |
